# Review of the *Berosus* Leach of Venezuela (Coleoptera, Hydrophilidae, Berosini) with description of fourteen new species

**DOI:** 10.3897/zookeys.206.2587

**Published:** 2012-07-06

**Authors:** Adriana Oliva, Andrew E. Z. Short

**Affiliations:** 1 Museo argentino de Ciencias naturales, Av. A. Gallardo 470, C1405DJR Buenos Aires, Argentina; 2 Division of Entomology, Biodiversity Institute and Department of Ecology & Evolutionary Biology, University of Kansas, 1501 Crestline Drive, Suite 140, Lawrence, KS 66045, USA

**Keywords:** Coleoptera, Aquatic beetles, South America, Guiana Shield

## Abstract

The species of the water scavenger beetle genus *Berosus* Leach occurring in Venezuela are reviewed. Thirty-six species are recorded, including fifteen new species, fourteen of which are described here as new: *Berosus aragua* **sp. n.**, *Berosus asymmetricus* **sp. n.**, *Berosus capanaparo* **sp. n.**, *Berosus castaneus* **sp. n.**, *Berosus corozo* **sp. n.**, *Berosus ebeninus* **sp. n.**, *Berosus garciai* **sp. n.**, *Berosus humeralis* **sp. n.**, *Berosus jolyi* **sp. n.**, *Berosus llanensis* **sp. n.**, *Berosus megaphallus* **sp. n.**, *Berosus ornaticollis* **sp. n.**, *Berosus repertus* **sp. n.**, and *Berosus tramidrum* **sp. n.** The fifteenth new species, known from a single female, is left undescribed pending the collection of males. Twelve species are recorded from Venezuela for the first time: *Berosus ambogynus* Mouchamps, *Berosus consobrinus* Knisch, *Berosus elegans* Knisch, *Berosus geayi* d’Orchymont, *Berosus ghanicus* d’Orchymont, *Berosus guyanensis* Queney, *Berosus holdhausi* Knisch, *Berosus marquardti* Knisch, *Berosus olivae* Queney, *Berosus reticulatus* Knisch, *Berosus wintersteineri* Knisch, and *Berosus zimmermanni* Knisch.

## Abstract (es)

Se revisan las especies del género *Berosus* Leach (Coleoptera: Hydrophilidae) de Venezuela. Se citan 36 especies, incluyendo 15 nuevas, 14 de las cuales se describen aquí como nuevas especies: *Berosus aragua* **sp. n.**, *Berosus asymmetricus* **sp. n.**, *Berosus capanaparo* **sp. n.**, *Berosus castaneus* **sp. n.**, *Berosus corozo* **sp. n.**, *Berosus ebeninus* **sp. n.**, *Berosus garciai* **sp. n.**, *Berosus humeralis* **sp. n.**, *Berosus jolyi* **sp. n.**, *Berosus llanensis* **sp. n.**, *Berosus megaphallus* **sp. n.**, *Berosus ornaticollis* **sp. n.**, *Berosus repertus* **sp. n.**, and *Berosus tramidrum* **sp. n.** La especie restante, conocida a través de una única hembra, se deja sin describir mientras se espera colectar machos. Se citan 12 especies nuevas para Venezuela: *Berosus ambogynus* Mouchamps, *Berosus consobrinus* Knisch, *Berosus elegans* Knisch, *Berosus geayi* d’Orchymont, *Berosus ghanicus* d’Orchymont, *Berosus guyanensis* Queney, *Berosus holdhausi* Knisch, *Berosus marquardti* Knisch, *Berosus olivae* Queney, *Berosus reticulatus* Knisch, *Berosus wintersteineri* Knisch, y *Berosus zimmermanni* Knisch.

## Introduction

*Berosus* Leach, 1817, one of five genera that comprise the tribe Berosini, is the largest genus of Hydrophiloidea with 273 species distributed worldwide (Hansen 1999, Short and Fikáček 2011). It is one of the most commonly collected groups of water scavenger beetles in the world as well as one of the most recognizable due to their typical “hunchbacked” appearance. All known species are aquatic, and generally good swimmers. They inhabit a range of “traditional” aquatic habitats, with most species occurring in lentic situations such as ponds and marshes. However, some species are known from other habitats such as stream margins and the slack waters of rivers. In the present work we describe the first species known in part from seepage-like habitats (*Berosus asymmetricus* sp. n.). The South American *Berosus* were comprehensively revised by Oliva (1989), with a significant addition four years later (Oliva 1993). Since then, a handful of additional species have been described, usually one at a time as they became known (e.g. Oliva 1995, 1998, 2002; Queney 2006). Up to now, 85 species were known to occur in continental South America, not counting species *incertae sedis*.

In the present work, we raise the total number of species known from Venezuela from 9 to 36, including the description of 14 new species. For comparison, that is twice the eighteen species that are known from all of North America.

## Materials and methods

More than 2400 specimens of *Berosus* from Venezuela were examined for this study. A large portion of this material was the result of recent survey efforts for aquatic insects in Venezuela. In addition to this newly collected material, we examined the large holdings of *Berosus* from the US National Museum of Natural History, and the two largest insect collections in Venezuela, the Universidad Central de Venezuela and the Universidad del Zulia. A few additional incidental collections were also examined.

**Institutional abbreviations are as follows:**

MACN Museo argentino de Ciencias naturales, Buenos Aires, Argentina (A. Roig-Alsina)

MALUZ Museo de Artrópodos, Universidad del Zulia (J. Camacho, M. García)

MIZA Museo del Instituto de Zoología Agrícola, Universidad Central de Venezuela (L. Joly)

MSUC Michigan State University Collection (A. Cognato, G. Parsons)

MTEC Montana State University (M. Ivie)

NHMW Naturhistorisches Museum, Wien (M. Jäch, A. Komarek)

NMPC National Museum, Praha (M. Fikáček).

SEMC Biodiversity Institute, University of Kansas, Lawrence, USA (A. Short)

USNM United States National Museum, Washington DC, USA (W. Steiner)

Specimens were examined using an Olympus SZX16 microscope to 100× magnification. Genitalia and abdominal ventrites were dissected from the specimen, cleared in cold KOH for 24 hours, and examined on temporary slides in glycerin. Photographs of these structures were taken using a DP-72 Olympus camera mounted on the abovementioned scope. Dorsal and lateral habitus photographs were taken with a Visionary Digital imaging system. All final images were created by stacking multiple individual photographs from different focal planes using the software CombineZ. Specimens listed without an explicit depository are divided between SEMC, MIZA, and MALUZ.

## Morphology and taxonomic characters of Venezuelan *Berosus*

**Figure 1.**
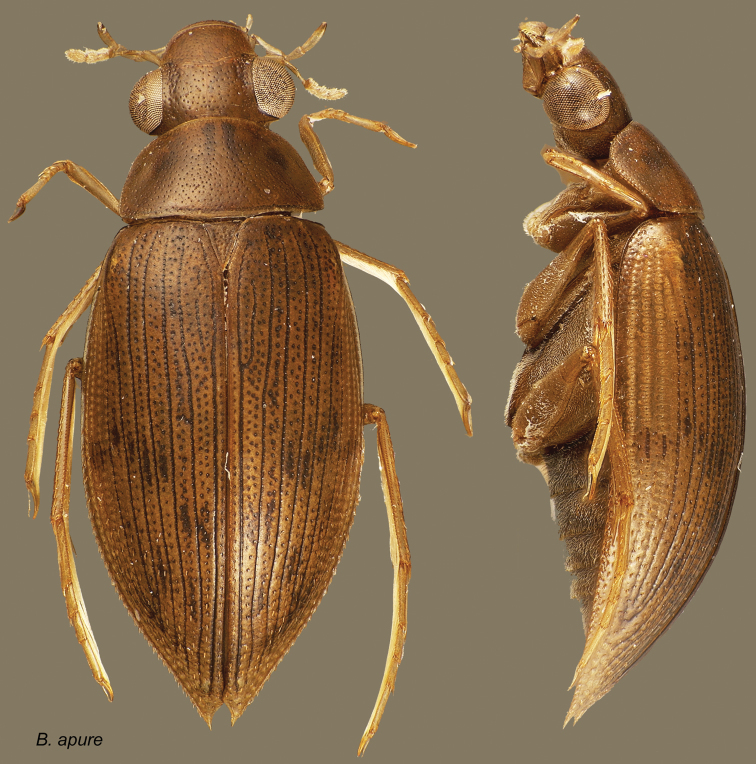
*Berosus apure*. Dorsal and lateral habitus.

**Figure 2.**
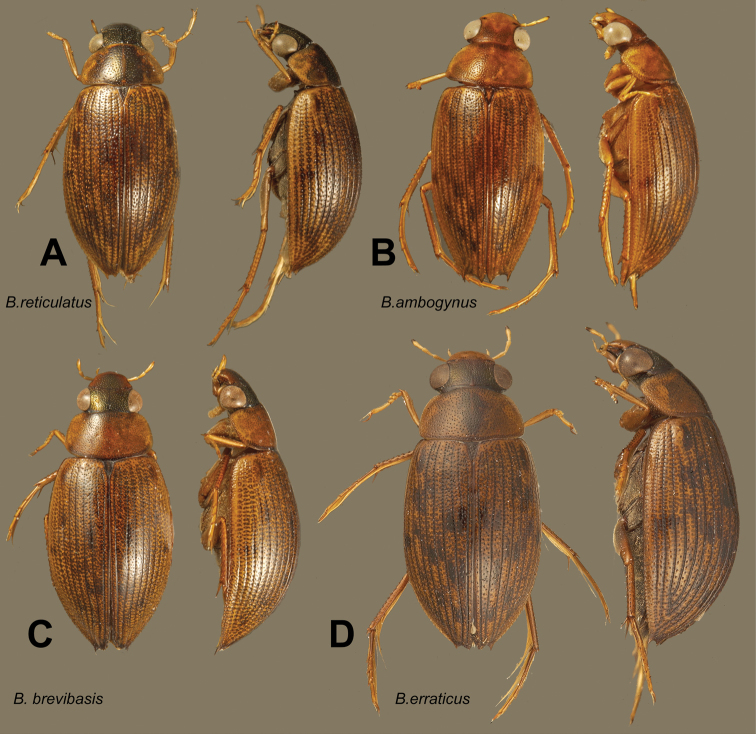
Dorsal and lateral habitus views of *Berosus* spp. **A** *Berosus reticulatus* **B** *Berosus ambogynus* **C** *Berosus brevibasis* **D** *Berosus erraticus*.

**Color:** Most species of *Berosus* have a brown dorsum, usually with a few darker maculae. The appearance may vary according to the age of the specimens (Oliva 1989), and in particular, melanic areas may appear reddish or black in individuals of the same species (Fig. 5). Most species have darkened areas as follows: dorsum of head; a median spot on the pronotum; the scutellum; on each elytron, two small sutural spots, one behind the other, between these a spot on the fifth interstria; a humeral spot on the sixth interstria; behind this, a spot on the seventh; a lateral spot corresponding to the stridulatory patch on the inner surface of the elytron, usually on the ninth and tenth interstriae; diffuse posterior spots on the third, fifth and seventh interstriae (Figs 2A, 14C, 23A, 25C). When the head is melanic, it usually has a strong metallic luster (Figs 2A, 14C, 23A). Variants of the color pattern are described with reference to the precedent, such as dorsum of head testaceous (Figs 1, 2A–C). In a few cases, the frons is melanic and the clypeus testaceous (Fig. 2D). The elytra may bear supplementary spots (Figs 5, 16) or lack some of them (Figs 6B, 19A).

**Sculpture:** The elytra bear ten rows of sunken punctures, plus a short basal stria between the first and the second ones. The striae are counted from the elytral suture outwards. The interstriae may be flat or convex, rarely carinate. The elytral apices may be produced (Figs 1, 24) or emarginate (Figs 2, 22), and may exhibit sexual dimorphism. The sculpture is termed “coarse”, “dense”, or “irregular” (“primitive sculpture” of Oliva, 1989) when the pronotal punctures are 3–4 times the size of an ommatidium, spaced by little less than their diameter so that they form wavy lines, but these lines often wider apart than the diameter of a puncture (Fig. 5). Interspersed with the punctures are micropunctures about one-quarter of the size of the latter. The scutellum often has the same type of sculpture. The elytra bear deep, wide striae, with the outer margin a little lower than the inner one. The punctures on the striae are coarse and thick, usually finer or shallower (or both) towards the elytral base and apex, on the outer striae overflowing outwards (Fig. 5). The interstriae bear punctures a little finer than the strial ones, uniseriate on the narrower interstriae, multiseriate on the wider ones (Fig. 5). Sculpture is termed “very coarse” (“hypertrophied sculpture” of Oliva, 1989) when the pronotal punctures are about six times the size of an ommatidium, and the strial punctures even larger (Figs 10, 14 A–D) The interstrial punctures are fine, sometimes obsolete. Sculpture considered “fine” appears as in Fig. 1.

**Male genitalia:** Trilobed, compressed, with open basal piece. Parameres articulated to basal piece. Median lobe without lateral appendices. Some species have appendices on the parameres (Fig. 21). Oliva (1989) found that the South American species present two different types of male genitalia. In one, the parameres are parallel to each other; in the other, the ventral margins of the parameres form a dihedrous angle. Each type presents several subtypes which were used to determine species-complexes instead of following the traditional division in subgenera. No South American species could be considered consubgeneric with the typical species of the subgenus, which has a very simple model of male genitalia (Schödl 1992). Thus, Oliva (1989) arranged the Neotropical species into species groups rather than following the traditional subgeneric divisions (see Oliva 1989 for more discussion).

## Checklist of Venezuelan *Berosus* by species complex

Complex definitions follow Oliva (1989). Asterisks (*) denote new records for Venezuela.

***Berosus alternans* complex**

Parameres forming a dihedral angle, narrowed to a blunt or rounded apex. Median lobe weakly curved, apex not strongly thickened. Metaventral process with posterolateral angles not produced, posterior angle carinate. First ventrite without lateral depressions. Protarsus of male with adhesive soles on the two basal tarsomeres. Elytra with spine-like hairs or not. Dorsal sculpture fine, if coarse micropunctation sparse or absent then elytral striae fine, sometimes the tenth stria reduced to a row of punctures on anterior half.

*Berosus aragua* sp. n. (Venezuela).

***Berosus auriceps* complex**

Parameres parallel to each other, acuminate (apex may be blunt). Median lobe thick, straight or weakly curved, not thickened at apex. Protarsus of males with soles on basal tarsomere only. Metaventral process long, not broadened at the level of the posterolateral angles, which are rounded, not produced into laminae. First ventrite carinate on anterior half, without lateral depressions, but sometimes with small glabrous areas in place of the latter. Elytra with a few small spine-like hairs on the posterior third of the outer interstria. Dorsal sculpture coarse. Body shape moderately depressed. Humeral humps prominent. All known species with strong metallic luster on dorsum of head, and often on pronotal spot.

*Berosus humeralis* sp. n. (Venezuela).

*Berosus ornaticollis* sp. n.(Venezuela).

***Berosus chalcocephalus* complex**

Male genitalia compressed. Parameres forming a dihedral angle, long, with apical portion dilated, in several species with inner membranous appendices. Median lobe weakly curved, weakly dilated at apex. Protarsus of males with soles on the two basal tarsomeres. First ventrite carinate only between posterior coxae, with small, shallow lateral depressions. Elytra with spine-like hairs on posterior third of elytra, in some species restricted to eleventh interstria. Dorsal sculpture moderately coarse and dense, punctures usually round; micropunctures dense, about one-fourth the size of the punctures. Body shape moderately convex, elongate. Elytral apices usually produced, or emarginate, or both.

*Berosus pallipes* Brullé, 1841 (Argentina, Brazil, Chile, Uruguay, Venezuela).

***Berosus corumbanus* complex**

Male genitalia cylindrical to compressed. Parameres forming a dihedral angle, acuminate, clearly divided lengthwise into a membranous area and a more strongly sclerotized one. Median lobe strongly swollen at apex, usually spindle-shaped. First ventrite carinate between metacoxae or for a stretch behind the latter, without lateral depressions. Elytra with or without spine-like hairs. Lateral margins of ventrites 1–4 entire, that of fifth ventrite often serrate. Dorsal sculpture fine or moderately coarse.

*Berosus castaneus* sp. n. (Venezuela).

*Berosus geayi* d’Orchymont, 1937* (Brazil, French Guiana, Venezuela).

*Berosus pluripunctatus* Mouchamps, 1963 (Brazil, Venezuela).

***Berosus holdhausi* complex**

Male genitalia weakly compressed. Parameres parallel, acuminate. Median lobe strongly curved, forming the shape of an “s”, strongly swollen at apex, the swelling spindle-shaped or blunt. Protarsus of males not or hardly swollen at base, without soles of specialized hairs. Dorsal sculpture coarse or very coarse. Metaventral process short, wide, broadened at the level of the strongly raised, rounded posterolateral angles. First ventrite carinate on most (usually all) of its length, without lateral depressions. Fifth ventrite raised at each side of the wide, shallow apical notch, produced at the bottom into a pair of sharp teeth. Lateral margins of ventrites crenulate in most species. Elytra without spine-like hairs. Body shape convex, in dorsal aspect usually wide, sometimes elongate and sturdy; humeral humps always prominent. Dorsal color varying for each species (Fig. 14A–D).

*Berosus consobrinus*Knisch, 1921* (Brazil, Venezuela).

*Berosus corozo* sp. n. (Venezuela).

*Berosus ebeninus* sp. n.(Venezuela).

*Berosus holdhausi* Knisch, 1921* (Argentina, Bolivia, Brazil, Venezuela).

*Berosus marquardti* Knisch, 1921* (Brazil, Venezuela).

*Berosus tramidrum* sp. n.(Venezuela).

*Berosus wintersteineri* Knisch, 1921* (Brazil, Venezuela).

*Berosus zimmermanni* Knisch, 1921* (Argentina, Brazil, Paraguay, Venezuela).

***Berosus obscurifrons* complex**

Male genitalia elongate, weakly compressed. Parameres acuminate, forming a dihedral angle. Median lobe weakly curved, weakly swollen at apex. Protarsus of males with soles on two basal tarsomeres. Metaventral process short, very wide, with posterolateral angles produced into small laminae. First ventrite carinate only between metacoxae, with small, shallow lateral depressions. Fifth ventrite with deep apical notch, produced at bottom into a pair of teeth. Elytra with spine-like hairs on interstria eleventh. Dorsal sculpture fine, without micropunctation. Body shape convex, elongate, with sides strongly convex, more prominent that the humeral humps. Dorsum without metallic luster in the known species.

*Berosus elegans* Knisch, 1921* (Brazil, Venezuela).

***Berosus patruelis* complex**

Male genitalia subcylindrical. Parameres acuminate, parallel. Median lobe thick, weakly curved, with a dorsal ridge that may be limited to the apex, and may be raised into a half-disc or a point. First ventrite carinate only between metacoxae, with or without lateral depressions. Elytra with spine-like hairs on all the interstriae on posterior half. Protarsus of male with soles on the two basal tarsomeres, which may be very weakly swollen. Eyes remarkably large, sometimes more so in males. Dorsal sculpture coarse, but with fine elytral striae. Shape depressed, in dorsal aspect narrow, with prominent humeral humps.

*Berosus patruelis* Berg, 1885 (Argentina, Bolivia, Brazil, Paraguay, Venezuela).

*Berosus repertus* sp. n. (Venezuela).

***Berosus reticulatus* complex**

Male genitalia compressed. Parameres elongate, narrowed or rounded at apex, forming a dihedral angle. Median lobe weakly curved, with apex weakly swollen, spindle-shaped, rarely simply truncated. Protarsus of male with soles on the two basal tarsomeres, which are moderately or strongly swollen. Elytra with spine-like hairs on all the interstriae. All the known species with elytral apices bispinous. Metaventral process variable, if wide not broadened at the level of the posterolateral angles; posterior angle not raised in known species. First ventrite carinate only between metacoxae, with small, deep lateral depressions.

*Berosus ambogynus* Mouchamps, 1963* (Bolivia, Brazil, Venezuela).

*Berosus brevibasis* Oliva, 1989(Brazil, Venezuela).

*Berosus capanaparo* sp. n. (Venezuela).

*Berosus erraticus* Mouchamps, 1963 (Argentina, Bolivia, Brazil, Paraguay, Uruguay, Venezuela).

*Berosus ghanicus*d’Orchymont, 1941* (Brazil, Guyana, Venezuela).

*Berosus reticulatus* Knisch, 1921* (Argentina, Brazil, Paraguay, Venezuela).

***Berosus sticticus* complex**

Male genitalia compressed. Parameres parallel, acuminate. Median lobe strongly or weakly curved, weakly to moderately swollen at apex, which is usually spindle-shaped. Protarsus of males with soles on the two basal tarsomeres, which are weakly to moderately swollen. Elytra without spine-like hairs. First ventrite carinate behind metacoxae, without lateral depressions. Metaventral process variable, usually but not always with posterolateral angles produced, posterior angle raised into a lamina or not. Dorsal sculpture varying from coarse to fine, with or without micropunctation. Size small (body length rarely exceeding 4 mm). Body shape moderately convex to depressed, in dorsal aspect oval, rarely narrow. All the known species without metallic luster on dorsum.

*Berosus asymmetricus* sp. n. (Venezuela).

*Berosus festivus* Berg, 1885 (Argentina, Brazil, Guyana, Uruguay, Venezuela).

*Berosus garciai* sp. n. (Venezuela).

*Berosus guyanensis* Queney, 2006* (French Guiana, Venezuela).

*Berosus jolyi* sp. n. (Venezuela).

*Berosus llanensis* sp. n. (Venezuela).

*Berosus olivae* Queney, 2006* (French Guiana, Venezuela).

***Berosus subtilis* complex**

Male genitalia moderately compressed. Parameres forming a dihedral angle, varying in shape, but never simply rounded. Median lobe swollen at apex, the shape of the swelling varying. Protarsus of males with soles on the two basal tarsomeres, which are strongly swollen. First ventrite carinate only between metacoxae, with large, rounded lateral depressions. Elytra with spine-like hairs on all the interstriae. Elytral apices produced or emarginate, or both. Shape depressed, slender, elongate. Dorsal sculpture coarse to fine, moderately dense to sparse, often reticulate in parts, at least in females. Dorsum without metallic luster in known species.

*Berosus apure* Oliva, 2002 (Venezuela).

*Berosus ruffinus* d’Orchymont, 1946 (Bolivia, Brazil, Venezuela).

***Berosus truncatipennis* complex**

Male genitalia cylindrical, large. Parameres more or less acuminate, forming a dihedral angle, widely separated dorsally, with internal membranous appendices. Median lobe simple, with spindle-shaped apex, or complex. Protarsus of males with soles on the two basal tarsomeres, which are strongly swollen. Elytra with spine-like hairs on all the interstriae or only on the eleventh. First ventrite carinate between metacoxae or for a stretch behind, with large, deep, rounded lateral depressions in most species (rarely small, shallow). Dorsal sculpture coarse to moderate, pronotal punctures usually round; micropunctation moderate to fine, usually dense. Dorsum without metallic luster in the known species. Several species with remarkable sexual dimorphism in the fifth ventrite: in females, the apex is evenly and broadly emarginated, while in males the emargination is deep and horseshoe-shaped.

*Berosus megaphallus* sp. n. (Guyana, Venezuela).

*Berosus truncatipennis* Castelnau, 1840 (Argentina, Bolivia, Brazil, ?Cuba, Guatemala, Mexico, Nicaragua, Panama, Paraguay, Venezuela).

***Incertae sedis*:**

Sp. A (Venezuela).

## Species treatments

### Berosus ambogynus

Mouchamps, 1963

http://species-id.net/wiki/Berosus_ambogynus

Figs 2B , 3A , 26

Berosus (Enoplurus) ambogynus Mouchamps, 1963: 123.
Berosus ambogynus Mouchamps: Oliva (1989: 164).

#### Material examined

**(40):** **VENEZUELA:** **Amazonas State:** ca. 7 km S. Samariapo, 5°10.900'N, 67°46.078'W, 95 m, roadside pond; 15.i.2009, leg. Short, Miller, García, Camacho & Joly, VZ09-0115-02X (1 ex., SEMC). **Apure State:** ca. 6 km S. Rio Cinaruco, 6°30.900'N, 67°32.604'W, 68 m, morichal and marsh, 8.i.2006, leg. Short, AS-06-019 (6 exs., SEMC, MIZA); same locality but 17.i.2009, leg. Short, Miller, & Camacho, VZ09-0117-01X (6 exs., SEMC); 44 km N. Rio Capanaparo, 7°20.175'N, 67°43.868'W, 49 m, marsh at sand dunes, 11.ix.2007, leg. Short, AS-07-004 (2 exs., SEMC). **Bolívar State:** between Caicara & Los Pijiguaos, 7°20.992'N, 66°17.904'W, 62 m, pond and lagoon, 11.i.2009, leg. Short, Miller & García, VZ09-0111-02X (2 ex., SEMC); ca. 20 km E. Maripa, 7°26'23.2"N, 64°57'5.6"W, 45 m, grassy flooded area, 5.viii.2008, leg. Short & García, AS-08-074 (1 ex., SEMC). **Guárico State:** 8°6.226'N, 66°26.228'W, 52 m UCV San Nicolasito Field Station, Rio Aguaro, 10.i.2009 leg. Short, Miller, Joly, García & Camacho, VZ09-0110-01A (22 exs., SEMC, MIZA, MALUZ). Representative specimen will be deposited in NHW, NMPC, and USNM.

#### Distribution.

Bolivia (Tarija), Brazil (Amazonas, Pará) and Venezuela (Amazonas, Apure, Bolívar, Guárico).

#### Remarks.

While not common, this species has been collected in a variety of habitats on both sides of the Orinoco. Most collections were made in standing waters, including both temporary and likely permanent roadside ponds and marshes. Other collections were made along the margins of morichals, and the largest series was taken in a shallow slack-water pool of a small river.

### Berosus apure

Oliva, 2002

http://species-id.net/wiki/Berosus_apure

Fig. 1

Berosus apure Oliva, 2002: 98.

#### Material examined 

**(6):** **VENEZUELA: Guárico State:** Corozo Pando (8 km N.), 17–18.vi.1984, blacklight, leg. F.W. Eiland & V. Linares (4 exs., USNM, SEMC); c. 65 km S. Las Mercedes, 8°31.705'N, 66°22.602'W, 145 m, large marsh, 6.vii.2010, leg. Short, Tellez, Camacho, & Arias, VZ10-0706-03B (2 exs., MIZA, SEMC).

**Figure 3.**
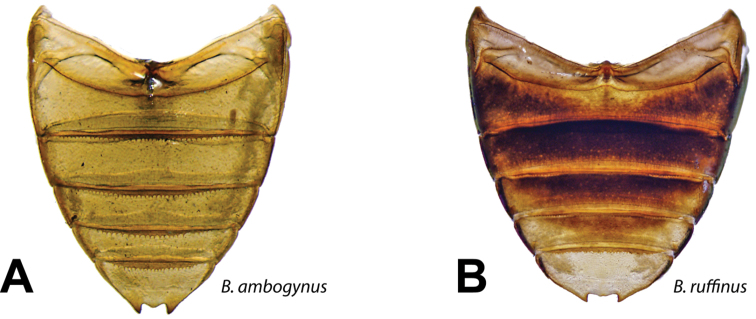
Abdomens of *Berosus* spp. **A** *Berosus ambogynus* **B** *Berosus ruffinus*.

#### Distribution.

Venezuela (Apure, Guárico).

#### Remarks.

This species was recently described from a series of specimens from central Apure State, slightly west of the localities reported here from Guárico State. All known localities are in the lowland savannahs of the central Llanos region.

### Berosus aragua

Oliva & Short sp. n.

urn:lsid:zoobank.org:act:B9E43C12-AACE-4512-A970-AD143DC2739C

http://species-id.net/wiki/Berosus_aragua

Fig. 5

#### Type material.

**Holotype** (male): “VENEZUELA: Anzoategui State/ 9°17'16.3"N, 64°13'39.1"W, 274 m/ Transect #1; 15.viii.2009/ temporary rain pond/ leg. R. Cordero; VZ09-0815-12A”, “[barcode]/ SEMC0889759/ KUNHM-ENT”, “HOLOTYPE/ BEROSUS/ aragua sp. n./ des. Oliva & Short 2010” (MIZA). **Paratypes (50): VENEZUELA: Anzoátegui State:** Transect 1, 09°07'19.7"N, 64°11'11.4"W, 216 m, 13.viii.2009, temporary rain pond on clay road, leg. R. Cordero, VZ09-0813-08A (1 ex., SEMC); same data as holotype (4 exs., SEMC); Transect 1, 09°17'58.0"N, 64°13'39.2"W, 276 m, 15.viii.2009, pond with shrubs and grass, leg. R. Cordero, VZ09-0815-13A (1 ex., SEMC). **Aragua State:** Cagua, 28.vi.1961 (23 exs., MSUC, SEMC, MIZA, MALUZ). **Falcón State:** E. Dabajuro, river at bridge crossing on Rt. 3, 8.vii.2009, 11°02.305'N, 70°38.467'W, 98 m, gravel/sand margins of river and associated sidepools, leg. Short, Gustafson, & Inciarte, VZ09-0708-01A (6 exs., SEMC); same data but leg. Sites & Shepard, VZ09-0708-01B (4 exs., SEMC); ca. 18 km E. Urumaco, Lagoon along Rt. 3, W. of Coro, 11°14.228'N, 70°05.312'W, 79 m, marginal, vegetated areas of lagoon, 8.vii.2009, leg. Short, Gustafson, García, Camacho, & Inciarte, VZ09-0708-02A (2 exs., SEMC); SE Tocopero, 10.vii.2009, leg. Short et al., muddy pool in roadside ditch, VZ09-0710-03C (1 ex., MIZA). **Guárico State:** 8 km N. Corozo Pando, 11.vi.1984, leg. F.W. Eiland (4 exs., USNM); same locality but 20-21.vi.1984, blacklight, leg. F.W. Wiland & V. Linares (3 exs., USNM); same data but 17–18.vi.1984 (1 ex., USNM).

#### Diagnosis.

This species appears to be closely related to *Berosus alternans* Brullé, 1841 (Argentina), by the shape of the male genitalia and the metaventral process not produced at the posterolateral angles. *Berosus aragua* has a much longer row of hairs on the paramera and a slightly shorter basal piece. Additionally, this new species has more deeply impressed elytral striae; the mesoventral process has the posterior tooth rounded, the posterior angle of the metaventral process is not raised and the apical notch of the fifth ventrite is produced into a pair of acute teeth. In *Berosus alternans*, the elytral striae are reduced to rows of punctures in anterior half, the posterior angle of the mesoventral process is not rounded, the posterior angle of the metaventral process is carinate and the apical notch of the fifth ventrite is produced into a pair of rounded (not acute) projections.

**Description.** Body length 3.1–4.5 mm (holotype: body length: 3.7 mm; humeral width: 1.55 mm). Shape depressed, moderately elongate (Fig. 5). Labrum melanic, dorsum of head melanic with strong metallic luster. Pronotum testaceous with small medial melanic spot, divided by a wide median testaceous line. Scutellum dark. Elytra testaceous with small melanic spots, with additional spots extending between the humeral spot and the first sutural spot, in some specimens forming an oblique dark band. Venter of thorax reddish. Abdomen black (Fig. 5A, B). Maxillary palpi with distal palpomere darkened on apical half. Femora with pubescent portion darkened, glabrous portion testaceous.

Head densely punctate, punctures on clypeus 1–2 times as large as ommatidia, regularly spaced; punctures on frons 2–4 times as large as ommatidia, irregular, sometimes contiguous.

Pronotal punctures dense and coarse, with punctures on disc about the same size as those on frons, on posterolateral areas polygonal. Pronotum between punctures very sparsely micropunctate, shining. Scutellum coarsely punctate, shining. Elytral striae well-impressed, with small round punctures about the same size as pronotal ones, not overflowing outwards except on striae 4–6, the intervals between punctures lower than interstriae. Interstriae flat, wide, three times or more as wide as striae, the fourth, fifth and sixth slightly step-shaped at disc due to overflowing strial punctures; tenth weakly convex on anterior half; eleventh flat, but raised with respect to spaces between strial punctures. Inner interstriae with punctures about half the size of the strial ones, 1–2 seriated; outer interstriae with much finer punctures. Surface of elytra smooth and shining in males, reticulate in females. Elytral apices simple; spine-like hairs absent.

Mesoventral process with large curved anterior tooth pointed downwards and backwards, behind this the ventral margin concave, describing a quarter of circle; posterior angle rounded, less prominent than anterior tooth. Metaventral process narrow; posterolateral angles not produced, posterior angle not raised. First ventrite carinate between metacoxae and a little behind them. No lateral depressions. Ventrites 2–4 without carinate or teeth. Fifth ventrite with a wide, shallow apical notch, which is set medially with two distinct sharp teeth. Margins of all abdominal ventrites smooth.

Maxillary palpi short, apical palpomere about twice as long as penultimate, slender, subcylindrical. Basal pubescence on basal half of mesofemora and three-fifths of metafemora, limit convex towards apex. Protarsus of male with adhesive soles on the two basal tarsomeres, the first of which is thickened, as long as the second and third combined; fourth tarsomere thickened, as long as the first and second taken together. Claws slender, weakly arched, toothed at base.

Male genitalia laterally compressed (Fig. 5D). Basal piece two-fifths of total length. Parameres long, narrow, gradually acuminate, weakly curved towards the sternal side. Row of hairs on the parameres long, taking up about half the total length. In sternal view the parameres parallel-sided taken together, gently rounded distally, not broadened, with apices turned inwards. Median lobe shorter than parameres, subcylindrical, slender, straight.

#### Variation.

This species exhibits a wider range of color variation than typical for most other Venezuelan *Berosus*, ranging from very pale to very dark, although some of the more darkened specimens appear to be so due to specimen preservation. Most material from Falcón State, the dorsal coloration is very pale, with some specimens entirely lacking pronotal maculae.

Regardless, the apical abdominal and aedeagal morphology are identical in these various color morphs.

#### Etymology.

The name refers to the Venezuelan region of Aragua, one of the states where this species was found.

#### Distribution.

Venezuela (Anzoátegui, Aragua, Falcón, Guárico).

#### Remarks.

This species has been taken along the densely vegetated margins of ponds, in the gravel sidepools of rivers, and at lights.

### Berosus asymmetricus

Oliva & Short sp. n.

urn:lsid:zoobank.org:act:95A20A99-FF7C-4ED5-8799-4922114A6B73

http://species-id.net/wiki/Berosus_asymmetricus

Figs 6A , 7A , 27

#### Type material.

**Holotype** (male): “VENEZUELA: Bolivar State/ 6°35.617'N, 66°49.238'W, 80 m, Los Pijiguaos; 6.viii.2008/ leg. A.Short, M.García, L.Joly/ AS-08-076; morichal/rock outcrop”, “HOLOTYPE/ BEROSUS/ asymmetricus sp. n./ des. Oliva & Short 2010” (MIZA). **Paratypes (112): VENEZUELA: Amazonas State:** nr. Iboruwa, “Tobogancito”, 5°48.414'N, 67°26.313'W, 80 m, rock pool with detritus, 7.viii.2008; leg. Short, García, & Joly, AS-08-078 (2 exs., SEMC); ca. 15 km S. Puerto Ayacucho, 5°30.623'N, 67°36.109'W, 100 m, ‘rock pools et al.’ on granite outcrop, 14.i.2009, leg. Short, VZ09-0114-03B (1 ex., SEMC). **Bolívar State:** Same data as holotype (55 exs., MIZA, MALUZ, SEMC); same locality as holotype but 7.vii.2010, leg. Short, Tellez & Arias, rock pools, VZ10-0707-01A (24 exs., MIZA, SEMC); same locality as holotype but 8.vii.2010, leg. Short, Tellez, & Arias, rock pools, VZ10-0708-01A (25 exs., MIZA, SEMC); same locality but 8.vii.2010, leg. Short, Tellez & Arias, small stream on outcrop, VZ10-0708-01B (6 exs., SEMC).

#### Diagnosis.

The males of this species resemble *Berosus festivus* Berg in the thin carina in front of the apical notch. They differ in the unique asymmetrical genitalia and in the coarsely serrate edge of the mesoventral process.

#### Description.

Body length 2.1–2.6 mm; (holotype: total length: 2.3 mm; humeral width: 1.0 mm). Shape depressed. Head testaceous with a melanic triangle taking up a small part of the clypeus and the greater part of the frons. Pronotum testaceous with a strongly melanic band on the anterior margin behind the head; a pair of elongate paramedial discal spots; a less deeply melanic posterior band taking up one-third of the pronotum excepting the lateral margins. Scutellum dark. Elytra testaceous with small dark spots. Venter of thorax and abdomen dark brown in typical series. Maxillary palpi with distal palpomere darkened on apex. Femora with pubescent portion darkened, glabrous portion testaceous.

Punctures on clypeus fine and regular, coarser on frons, irregularly spaced, polygonal rather than round, rather sparse except along inner margin of the eyes, where they are contiguous. Pronotum with moderate-sized punctures (about twice the size of ommatidia), elliptical, with irregular spaces 1–4 times their diameter, which are shining, not micropunctate. Scutellum shining, with a few punctures similar in size to those on the pronotum. Elytral striae with round punctures, twice as large as those on the pronotum, contiguous, on the basal stria spaced by a whole diameter or a little more. Interstriae flat on elytral disc, not step-shaped, 1.5–2 times as wide as striae, sparsely and finely punctate (punctures smaller than those on pronotum); between punctures shining, not micropunctate. Interstriae 10 convex, overhanging the eleventh between humeral humps and stridulatory patch. Eleventh interstria flat, about 1.5 times as wide as the tenth. Spine-like hairs absent.

Mesoventral process with curved anterior tooth directed downwards and backwards; behind this, the ventral margin concave and irregularly serrate, in some specimens forming a small, acute second tooth; posterior angle weakly raised. Metaventral process rather wide, posterolateral angles raised into rounded lamellae, posterior angle carinate, not raised. First ventrite with carina thick between metacoxae, behind thin, reaching the posterior margin of the ventrite. Lateral depressions absent. Fifth ventrite with shallow apical notch, the bottom of the latter with two contiguous rounded teeth, in front of these a thin carina.

Maxillary palpi short and thick in both sexes, darkened at apex of fourth palpomere. Basal pubescence on half of mesofemora and three-fifths of metafemora, limit transverse. Protarsus of male short and thick, with basal tarsomere about 1.5 times as long as second, both weakly thickened, bearing small soles of adhesive hairs. Claws weakly arched.

Male genitalia (Fig. 7A): basal piece four-fifths of total length, three times as long as wide. Parameres with basal two-thirds encased in apical half of basal piece, asymmetrical. In lateral aspect, the unencased part of the parameres evenly curved towards the sternal side, describing nearly a quarter of a circle; apices narrowed. Row of hairs rather long. In tergal aspect, parameres strongly and regularly curved inwards. Median lobe longer than parameres, but strongly curved towards one side, subcylindrical, acuminate at the apex which is directed to one side.

#### Etymology.

The name alludes to the asymmetrical genitalia.

#### Distribution.

Venezuela (Amazonas, Bolívar).

#### Remarks.

This species, remarkable for its unusual genitalia, is apparently restricted to granite outcrops. All specimens have been collected in rock pools and small streams that occur on the bare rock of the numerous outcrops along the northwestern margin of the Guiana Shield region of Venezuela (Fig. 27). Several longer series have been collected in small seasonal streams that form on the outcrops which drain water during the wet season, as well as in small rock pools adjacent to the streams.

### Berosus brevibasis

Oliva, 1989

http://species-id.net/wiki/Berosus_brevibasis

Figs 2C , 4A

Berosus brevibasis Oliva, 1989: 165.

#### Material examined

**(28).** **VENEZUELA:** **Apure State:** ca. 6 km S. Rio Cinaruco, 6°30.900'N, 67°32.604'W, 68 m, morichal and marsh, 8.i.2006, leg. Short, AS-06-019 (5 exs., SEMC, MIZA), same locality but 17.i.2009, leg. Short, Miller, & Camacho, VZ09-0117-01X (1 ex., SEMC). **Bolívar State:** Los Pijiguaos, 6°35.617'N, 66°49.238'W, 80 m, rock outcrop/morichal, algal margin of morichal, leg. Short, García, Camacho, Miller, & Joly, VZ09-0112-01B (1 ex., SEMC); same locality but 15.ix.2007, leg. Short, HG-vapor light, AS-07-014 (1 ex., SEMC). **Guárico State:** 15 km S. Calabozo, 9-13.ii.1969, Lago de los Patos, leg. P. & P. Spangler (18 exs., USNM, SEMC); Corozo Pando, 12.ii.1969, leg. P. & P. Spangler (1 ex., USNM); UCV San Nicolasito Field Station, Rio Aguaro, 8°6.226'N, 66°26.228'W, 52 m, 10.i.2009, leg. Short, Miller, Joly, García, & Camacho, VZ09-0110-01A (1 ex., SEMC).

**Figure 4.**
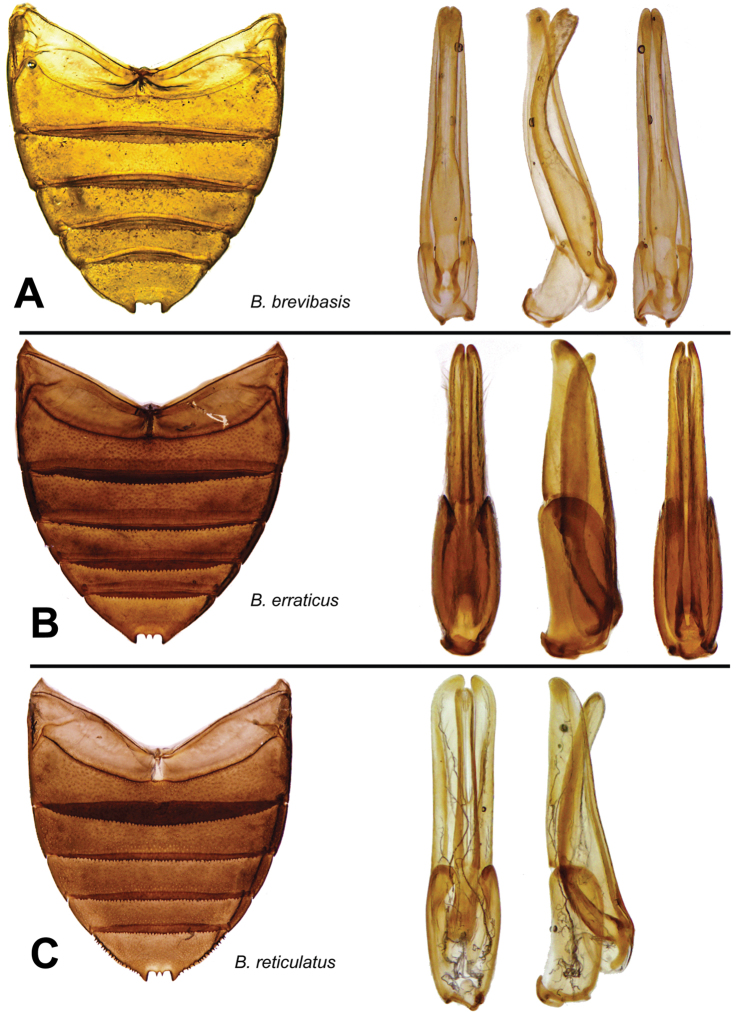
Abdomen and aedeagal views of *Berosus* spp. **A** *Berosus brevibasis* **B** *Berosus erraticus* **C** *Berosus reticulatus*.

**Figure 5.**
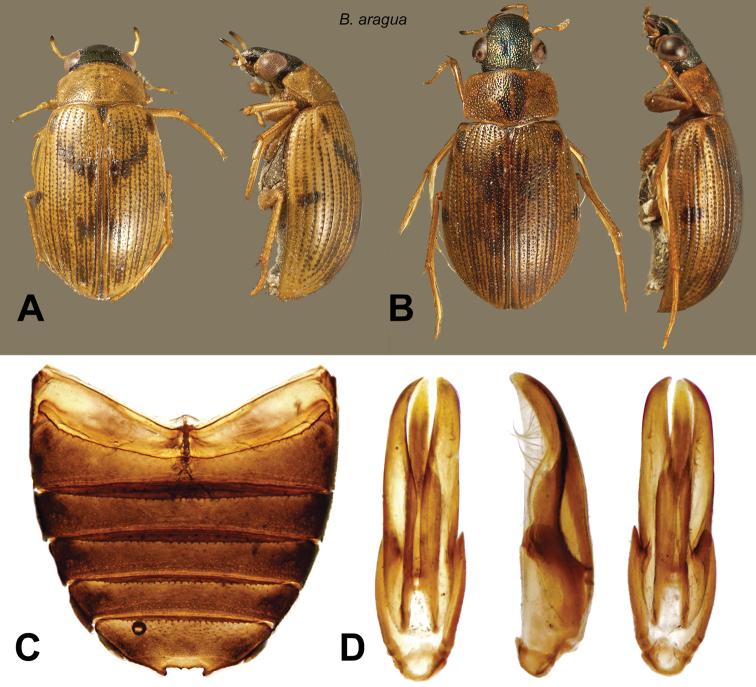
*Berosus aragua* sp. n. **A** dorsal and ventral habitus (lighter form) **B** dorsal and ventral habitus (darker form) **C** abdomen **D** aedeagus.

**Figure 6.**
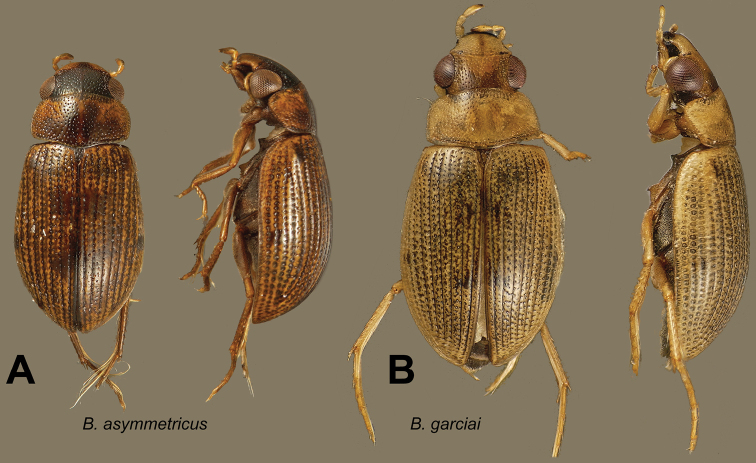
Dorsal and lateral habitus views of *Berosus* spp. **A** *Berosus asymmetricus* sp. n. **B** *Berosus garciai* sp. n.

**Figure 7.**
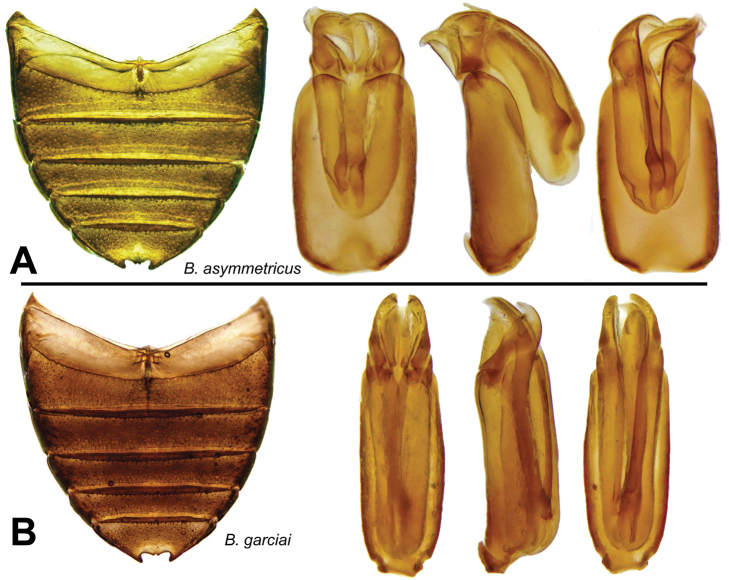
Details of *Berosus* spp. **A** *Berosus asymmetricus* sp. n., abdomen and aedeagus **B** *Berosus garciai* sp. n., abdomen and aedeagus

**Figure 8.**
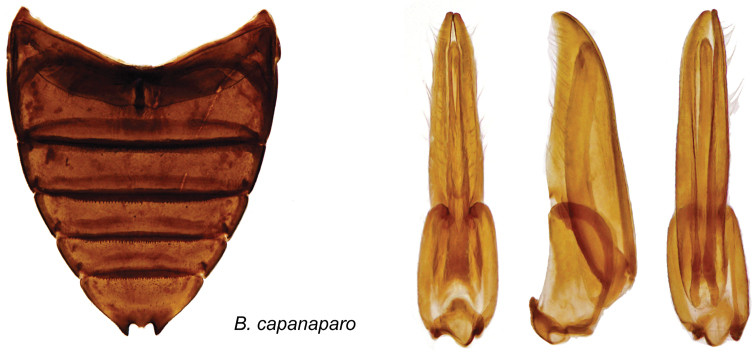
*Berosus capanaparo* sp. n, abdomen and aedeagus.

#### Distribution.

Brazil (Mato Grosso), Venezuela (Apure, Bolívar, Guárico).

**Remarks.** While Brazilian specimens were collected from gallery forest, the Venezuelan specimens were taken along morichals (rivers) and marshes in the Llanos and at a morichal along the northern edge of the Guiana Shield

### Berosus capanaparo

Oliva & Short sp. n.

urn:lsid:zoobank.org:act:0680E3E6-6A0E-4353-9FF1-42B4A56812A9

http://species-id.net/wiki/Berosus_capanaparo

Figs 8 , 28D

#### Type material.

**Holotype** (male): “VENEZUELA: Apure State/ 7°20.175"N, 67°43.868"W, 49m/ Medanos de la Soledad/ 17.i.2009; Short, Miller, Camacho/ VZ09-0117-02X; marshy area”, “[barcode]/ SEMC0877922/ KUNHM-ENT”, “HOLOTYPE/ BEROSUS/ capanaparo sp. n./ des. Oliva & Short 2010” (MIZA). **Paratypes (8):** **VENEZUELA: Apure State:** Same data as holotype (3 exs., SEMC, MIZA); Same locality but 11.ix.2007, leg. Short, AS-07-004 (5 exs., SEMC, MIZA).

#### Diagnosis.

Large species without metallic luster on dorsum. Abdomen black. Elytral apices bispinous, sutural angle not produced. Mesoventral process with curved anterior tooth, posterior angle strongly raised. First ventrite with lateral depressions: fifth with smooth margins, with apical notch produced at bottom into a pair of contiguous rounded teeth. Basal tarsomere of males about twice as long as second. Male genitalia compressed, parameres long, narrow, with short subapical row of hairs (Fig. 8).

#### Description.

Body length 5.0–6.7 mm (holotype: total length: 5.6 mm; humeral width: 2.2 mm). Shape elongate, humeral humps weakly prominent. Labrum and clypeus testaceous, frons melanic at base, in some specimens entirely black except on the angles between each eye and the frontoclypeal suture. Pronotum testaceous with a narrow central melanic spot divided by a testaceous median line. Scutellum diffusely melanic. Elytra testaceous with melanic spots. Venter of thorax reddish in typical series. Abdomen black. Maxillary palpi with fourth palpomere darkened on apical one-fourth. Femora with pubescent portion darkened, glabrous portion testaceous. Punctures on clypeus moderately dense, about the same size as ommatidia; on frons coarser and denser; micropunctation between punctures; frontal carina well-marked. Pronotal punctures about twice the size of an ommatidion, on disc spaced by 2–4 times their diameters, on sides contiguous. Elytra micropunctate between punctures, shining. Scutellum reticulate, with a few punctures. Elytral striae fine and well-incised, bearing punctures about the same size as the pronotal ones. Interstriae wide, flat, even the external ones, bearing punctures as coarse as the serial ones, 2–3 seriated. Elytral apices emarginate almost in a semicircle, with sutural angle not produced, parasutural tooth triangular, sharp. Spine-like hairs on all the interstriae on posterior third of elytra.

Mesoventral process laminar, with large, curved anterior tooth directed downwards and backwards, behind this the ventral margin straight, posterior angle strongly raised but much less so than anterior tooth. Metaventral process narrow, posterolateral angles produced into small triangular laminae; posterior angle not raised. First ventrite carinate only between metacoxae, with lateral depressions. Fifth ventrite with small apical notch raised at the sides, the bottom bearing a blunt bifid tooth (Fig. 8A). Maxillary palpi long, with fourth palpomere ensiform, longer than third; second palpomere as long as third and fourth together. Basal pubescence on three-fifths of meso- and metafemora, limit transverse. Protarsus of male with small adhesive soles on the two basal tarsomeres, which are weakly thickened, the basal one about twice as long as the following one. Claws slender, toothed at base.

Male genitalia (Fig. 8B) compressed: basal piece one-third of total length. Parameres long, narrow, weakly sinuate, with apices turned towards the sternal side, rounded; rows of hairs rather short, subapical. Median lobe shorter than parameres, subcylindrical, moderately thick, regular in thickness, weakly curved.

#### Etymology.

The name refers to the river Capanaparo, near which the new species was found.

#### Distribution.

Venezuela (Apure).

#### Remarks.

Both collecting events for this species were at the same unusual locality: a marsh and pools formed around a dune complex (the Médanos de la Soledad) in the middle of the Llanos region (Fig. 28D). One event each occurred during the dry and wet seasons

### Berosus castaneus

Oliva & Short sp. n.

urn:lsid:zoobank.org:act:A883F825-29FE-4140-96CD-7B877766CD58

http://species-id.net/wiki/Berosus_castaneus

Fig. 9

#### Type material.

**Holotype** (male): “VENEZUELA: Bolivar State/ 5°44'28.7"N, 61°30'54.3"W, E. of/ Kavanayen; 1.viii.2008; 1290 m/ leg. A. Short & M. Garcia; large/ vegetated marsh; AS-08-063”, “HOLOTYPE/ BEROSUS/ castaneus sp. n./ des. Oliva & Short 2010” (MIZA). **Paratypes (2): VENEZUELA: Bolívar State:** 5°37'53.8"N, 61°41'12.8"W, 1330 m, E. of Kavanayen, 1.viii.2008, leg. Short & García, small stream, AS-08-061 (1 male, SEMC); 48 km WSW Luepa, Salto Apanguao, 1220 m, 1.vii.1987, leg. M.A. Ivie, “stream-side wrack” (1 female, MTEC).

#### Diagnosis.

**Figure 9.**
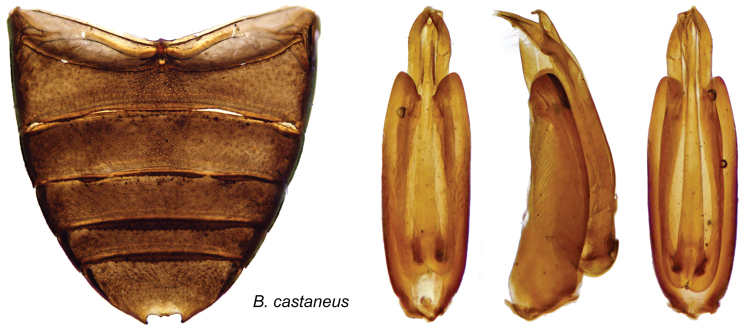
*Berosus castaneus* sp. n. abdomen and aedeagus.

This species appears close to *Berosus sinigus* Oliva, 1989 (Argentina, Brazil) and *Berosus hispidulus* Oliva, 1993 (Brazil), in the general shape of the genitalia and the color pattern. It differs from both in having spine-like hairs on the outer interstria only ant in the characters of the male genitalia, noticeably the very long basal piece (Fig. 9).

#### Description.

Body length 4.3 mm (holotype: total length: 4.3 mm; humeral width: 1.8 mm). Shape depressed. Labrum testaceous; clypeus testaceous with a very small dark median triangle medially, frons testaceous in lateral quarters, with central half darkened. Pronotum testaceous with a median melanic parallel sided stripe divided into two lateral halves by a thin testaceous median line. Scutellum dark brown. Elytra testaceous with dark spots in the usual pattern. Venter of thorax and abdomen very dark brown. Maxillary palpi yellow with apical segment darkened at apex. Femora with pubescent portion darkened, glabrous portion testaceous.

Clypeus with fine punctures (rather smaller than ommatidia), distance between punctures 1–2 times their diameter. Frons with punctures subequal in size to an ommatidion, distance between punctures 2–3 times their diameter. Maxillary palpi slender, not elongate. Pronotal disc with fine round punctures spaced by 2–4 times their diameter; surface shining, not microreticulate. Scutellum with dense punctures the same size as those on pronotum, surface shining. Elytral striae fine, on disc bearing punctures spaced by 1–2 times their diameter; outer striae more densely punctuated. Outer stria obsolete on apical fifth. Interstriae flat, with uniseriated punctures about the same size as those on striae, outer ones with obsolete punctures, on striae 9 and 11 a few larger punctures. Elytral apices separately rounded; with spine-like hairs on interstria 11 only.

Mesoventral process with small, straight anterior tooth directed posteroventrally, behind ventral margin weakly convex, then gradually depressed to posterior end which is not raised. Metaventral process narrow, finely carinate on front of medial depression, posterolateral angles produced into triangular laminae, posterior angle not raised. First ventrite carinate only between metacoxae, and without lateral depressions. Fifth ventrite with shallow apical notch, which is set with two triangular teeth. Meso- and metafemora with pubescence on basal two-thirds, the limit transverse, convex. Protarsus of male weakly thickened at base, with basal two tarsomeres expanded and with ventral pads, first tarsomere about twice as long as second. Claws weakly curved.

Male genitalia with basal piece extending three-quarters of total length. Parameres acuminate, unencased portion divided into a sternal membranous part, which has a weakly convex sternal margin, and a more strongly sclerotized tergal part, narrow, acuminate, straight.

Row of hairs rather short, extending along subapical third. Median lobe a little shorter than parameres, straight, acuminate at the apex.

#### Etymology.

The name alludes to the brown coloring of the dorsum, from the Latin *nux castanea*, “chestnut”, given adjectival form.

#### Distribution.

Venezuela (Bolívar).

#### Remarks.

All three known specimens were collected in the Gran Sabana region (at elevations of >1000 m): one each in a large marsh, a stream, and ‘stream-side wrack’.

### Berosus consobrinus

Knisch, 1921

http://species-id.net/wiki/Berosus_consobrinus

Fig. 10

Berosus (Berosus) consobrinus Knisch, 1921: 18.
Berosus consobrinus Knisch: Oliva (1989: 93); Oliva (1993: 203).

#### Material examined

**(23).** **VENEZUELA: Bolívar State:** 1 km S. San Francisco, 5°2.623'N, 61°6.083'W, 885 m, morichal stream/marsh, leg. Short & García, AS-08-068 (3 ex., MIZA); Chivaton Hotel, on rd. to Kavanayen, 1370 m, 28.vi.1987, leg. M.A. Ivie, at light (1 ex., MTEC). Guárico State: Corozo Pando (8 km N.), various dates between 11 and 21 June 1984, leg. F.W. Eiland & V. Linares (19 exs., MIZA, SEMC, USNM, MALUZ).

**Figure 10.**
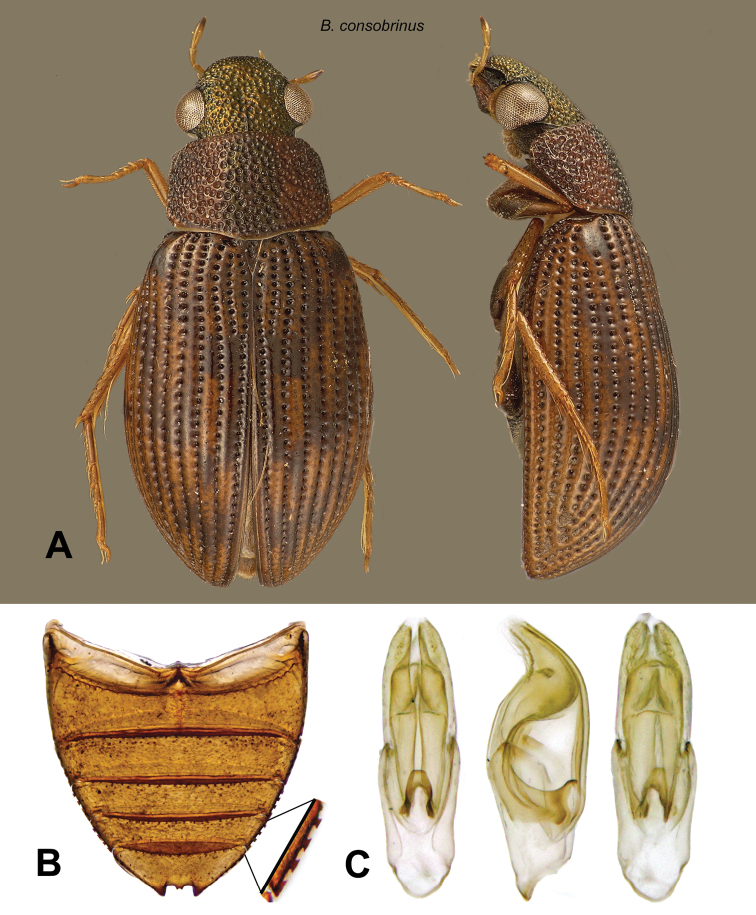
*Berosus consobrinus*. **A** dorsal and ventral habitus **B** abdomen **C** aedeagus.

#### Distribution.

Brazil (Mato Grosso), Venezuela (Bolívar, Guárico). Previously, only the type series was known.

#### Remarks.

This species was collected at two localities in the Gran Sabana, as well as a series from the central Llanos region.

### Berosus corozo

Oliva & Short sp. n.

urn:lsid:zoobank.org:act:2B4E0254-5C39-45FA-8ACA-8122D9DBBBF0

http://species-id.net/wiki/Berosus_corozo

Figs 14A , 15C

#### Type material.

**Holotype** (male): “VENEZUELA: Guarico/ Corozo Pando (8kmN.)/ 17 20-21 June 1984/ blacklight/ F.W. Eiland & V. Linares”, “HOLOTYPE/ BEROSUS/ corozo sp. n./ des. Oliva & Short” (MIZA). **Paratypes (31): VENEZUELA: Apure State:** Hato El Frio, Fundo Ceibote, 20.v.1975, leg. C. Rosales (21 exs., MIZA, SEMC, NHMW, NMPC); Fundo La Florida, Rio Quitaparo, 7°05'N, 68°26'W, 42 m, 11–14.vi.1999, leg. E. Osuna & A. Chacon (1 ex., MIZA). **Guárico State:** Same data as type (4 exs., USNM, SEMC); same data but 17–18.vi.1984 (5 exs., USNM, MALUZ ).

#### Diagnosis.

Moderate-sized species of the *Berosus holdhausi* complex, without metallic luster on dorsum, resembling *Berosus marquardti* Knisch, but without reddish hue on dorsum, with mesoventral process laminar, anterior tooth of this process straight, carina on first ventrite straight in lateral aspect and with median lobe of male genitalia subapically angular, apically narrow. This species is remarkable by the convex, short, broad body shape and the very coarse dorsal sculpture (Fig. 14A).

#### Description.

Body length: 3.6–4.3 mm. (holotype: total length: 4.3 mm; humeral width: 1.55 mm). Shape convex. Dorsum entirely testaceous, yellow in the type specimens, with base of frons, paramedial bands on pronotal disc and scutellum slightly darker. Elytra with small, weak darker spots on elytral disc and apical portion; the usual humeral and lateral spots are absent.

Pronotal punctures 3–6 times as large as ommatidia. Punctures on inner elytral striae about twice as large as pronotal ones, those on outer striae gradually larger. Inner interstriae about the width of striae, step-shaped, flat, shining, sparsely but deeply punctate. Outer interstriae 9 and 10 narrower than striae, weakly convex; interstria 11 weakly costate.

Mesoventral process in the shape of a short, strongly raised lamina with two teeth of equal height divided by a shallow semicircular notch; anterior tooth straight, directed posteriorly. Metaventral process short, wide, with posterolateral angles rounded and produced. First abdominal ventrite with thick, high carina along its entire length medially, straight in lateral aspect. Fifth ventrite strongly raised at the sides of small apical notch, which is medially set with two contiguous, diverging teeth. Lateral margins of abdominal ventrites finely crenulate. Meso- and metaventer with pubescence on basal two-thirds, distal limit briefly oblique. Protarsus of male with two basal tarsomeres bearing stiff hairs which are not expanded to form a pad.

Male genitalia (Fig. 15C) with basal piece about twice as long as wide, half of total length. Parameres strongly narrowed in apical third, apices weakly directed towards sternal side; sternal margin bearing a short row of long hairs on the subapical concave part. Median lobe shorter than parameres; in lateral aspect strongly curved towards the tergal side, subapically swollen with angular projection followed by a deep semicircular notch, narrowed apex directed towards the sternal side; in tergal aspect the subapical swelling spindle-shaped, with a median groove.

#### Etymology.

This species is named after the type locality, “Corozo Pando”.

#### Distribution.

Venezuela (Apure, Guárico).

#### Remarks.

Nothing is known about the biology of this species. Most specimens were collected at light.

### Berosus ebeninus

Oliva & Short sp. n.

urn:lsid:zoobank.org:act:98496051-3BCB-410A-A867-5F1966B88D51

http://species-id.net/wiki/Berosus_ebeninus

Figs 14B , 15A

#### Type material.

**Holotype** (male): “VENEZUELA: Bolivar State/ 5°34'29.8"N, 61°18'43.4"W, 1100 m, nr. Rio Sakaika; 2.viii.2008/ leg. A. Short & M. Garcia/ AS-08-067”, “[barcode]/ SM0829277/ KUNHM-ENT”, “HOLOTYPE/ Berosus/ ebeninus sp. n./ des. Oliva & Short 2010” (MIZA). **Paratypes (5)**: **VENEZUELA:** **Apure State:** Fundo La Florida, Rio Quitaparo, 7°05'N, 68°26'W, 42 m, 11–14.vi.1999, leg. E. Osuna & A. Chacon (2 exs., MIZA, SEMC); same data but 16-18.v.1999 (1 ex., MIZA). **Bolívar State:** Same data as holotype (1 female, SEMC); Cuchivero, 30 km SE of Caicara, 4.viii.1987, leg. S. & J. Peck, Woodland, UV light, SBP87-108 (1 male, SEMC).

#### Diagnosis.

This species keys to *Berosus consobrinus* Knisch, 1921 or *Berosus megillus* d’Orchymont in the key by Oliva (1993). The new species differs from the latter in having much finer punctation and the sides of the ventrites crenulate. From *Berosus consobrinus* it differs by the male genitalia and the extended melanization of the dorsum.

#### Description.

Body length: 3.6–3.9 mm. (holotype: total length: 3.9 mm; humeral width: 1.6 mm), Shape in dorsal aspect short and wide. Labrum, clypeus and frons dark brown to black with weak metallic sheen. Pronotum black in typical series, with metallic sheen, testaceous on lateral margins and, more extendedly, on anterior angles. Scutellum melanic. Elytra melanic, in the typical series deep black, shading into dark reddish brown at the sides and apices. Venter of thorax and abdomen dark reddish brown to nearly black. Maxillary palpi pale yellow with fourth palpomere darkened apically. Femora with pubescent and glabrous regions black and testaceous respectively. The remainder of the legs testaceous.

Punctures on clypeus about twice as large as ommatidia, contiguous, polygonal. Punctures on frons 3–4 times the size of an ommatidium, contiguous, polygonal, larger at the base of frons where there is a narrow median unpunctured line, not carinate. Pronotum with coarse (4–6 times an ommatidion), irregularly spaced (spaces equivalent to 1–2 diameters) punctures; surface coarsely micropunctate (micropunctures nearly as large as ommatidia). Lateral margins of pronotum serrate. Scutellum bearing a few punctures, rather smaller than those on pronotal disc. Elytral striae bearing punctures about the same size as those on pronotum, contiguous. Interstriae convex, narrower than striae, bearing punctures subequal in size as an ommatidium. Interstria 11 costate on anterior three-fourths, overhanging the elytral margin, not costate below humeral hump; the latter dentate. Elytral apices rounded; spine-like hairs absent. Mesoventral process laminar, with anterior tooth weakly curved, directed posteroventrally, behind this the free ventral margin concave, posterior angle weakly raised. Metaventral process wide, carinate before median depression, with posterolateral angles produced into rounded laminae, posterior angle finely carinate, not raised. Meso- and metafemora with pubescence on basal three-fifths and three-fourths respectively, its limit transversely oblique. Protarsus of male only slightly enlarged, the two basal tarsomeres slightly expanded and with a setal pad, which can hardly be said to form a sole. Claws weakly arched, dentate at base.

First ventrite medially carinate on most of its length, with carina faint to nearly absent at posterior margin. Fifth ventrite with small apical notch, set with two acute teeth medially. Margins of abdominal ventrites finely crenulate. Aedeagus (Fig. 15A) with basal piece about twice as long as wide, half of total length. Parameres gradually acuminate, curved, apices simply acuminate, directed towards the sternal side; row of hairs short, subapical. Median lobe longer than parameres, thick, weakly curved, abruptly swollen subapically, narrowed acuminate apex directed sternally.

#### Etymology.

The name derives from the Latin word *ebenus –i*, meaning “ebony”, and it alludes to the blackish dorsal coloring of this species.

#### Distribution.

Venezuela (Apure, Bolívar)

#### Remarks.

The specimens taken from the type locality were collected in a small, shallow marsh.

### Berosus elegans

Knisch, 1921

http://species-id.net/wiki/Berosus_elegans

Fig. 11

Berosus (Berosus) elegans Knisch, 1921: 8.
Berosus elegans Knisch: Oliva (1989: 167).

#### Material examined

**(693).** **VENEZUELA:** **Bolívar State:** Cuchivero, 30 km SE of Caicara, 4.viii.1987, leg. S. & J. Peck, woodland, UV light, SBP87-108 (1 ex., SEMC). **Guárico State:** 8 km N. Corozo Pando, 11.vi.1984, leg. F.W. Eiland (189 exs., USNM); same locality but 17–18.vi.1984, leg. F.W. Eiland & V. Linares, blacklight (25 exs., USNM); same locality but leg. F.W. Eiland & V. Linares, 20–21.vi.1984, blacklight (478 exs., USNM). Representative specimen will be deposited in MIZA, MALUZ, NHW, & NMPC.

**Figure 11.**
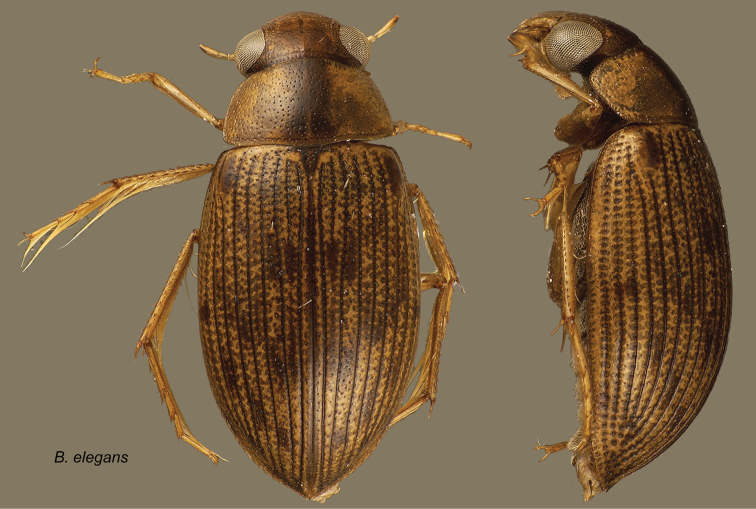
*Berosus elegans*. Dorsal and lateral habitus.

#### Distribution.

Brazil (Mato Grosso), Venezuela (Bolívar, Guárico).

#### Remarks.

It is interesting to note that while many hundreds of specimens were collected at one locality in the central Llanos region (most at lights), not one specimen was found from recent collecting efforts.

### Berosus erraticus

Mouchamps, 1963

http://species-id.net/wiki/Berosus_erraticus

Figs 2D , 4B

Berosus (Enoplurus) erraticus Mouchamps, 1963: 163.
Berosus erraticus Mouchamps: Oliva, 1989: 162.

#### Material examined

**(53).** **VENEZUELA:** **Anzoátegui State:** Transect 1, 9.33639°N, 64.19690°W, 300 m, Temporal rain pond in a dry forest, 12.viii.2009, leg. R. Cordero, VZ09-812-01A, (2 exs., SEMC); Transect 1, 09°21'03.1"N, 64°08'45.1"W, 327 m, Permanent pond with grass, 12.viii.2009, leg. R. Cordero, VZ09-812-02A, (1 ex., SEMC); Transect 1, 09°18'46.8"N, 64°07'11"W, 270 m, Permanent pond with canopy, near to corn crop, 12.viii.2009, leg. R. Cordero, VZ09-812-03A, (3 exs., SEMC); Transect 1, 09°17'06.2"N, 64°07'38.1"W, 251 m, Permanent pond under bridge, 12.viii.2009, leg. R. Cordero, VZ09-812-04A, (3 exs., SEMC); 09°19'41.4"N, 64°07'07.1"W, 289 m, temporary rain pond, 13.viii.2009, leg. Cordero, VZ09-813-07A (2 exs., SEMC); 09°07'19.7"N, 64°11'11.4"W, 216 m, temporary rain pond on the clay road, 13.viii.2009, leg. Cordero, VZ09-813-08A (1 ex., SEMC); 09°16'34.6"N, 64°13'39.3"W, 259 m, temporary pond by road, 14.viii.2009, leg. Cordero, VZ09-814-12A (2 exs., SEMC); Transect 1, 09°18'41.2"N, 64°13'10.2"W, 278 m, Temporal pond aside the road, 14.viii.2009, leg. R. Cordero, VZ09-814-13A, (3 exs., SEMC); Transect 1, 09°16'00.1"N, 64°13'42.9"W, 256 m, Temporal pond in a crossroad, 15.viii.2009, leg. R. Cordero, VZ09-815-11A, (4 exs., SEMC); 09°17'16.3"N, 64°13'39.1"W, 274 m, temporary pond by road, 15.viii.2009, leg. Cordero, VZ09-815-12A (11 exs., SEMC, MIZA); 09°17'58.0"N, 64°13'39.2"W, 276 m, permanent pond with dry shrubs and grass, 15.viii.2009, leg. Cordero, VZ09-815-13A (2 exs., SEMC). **Bolívar State:** Cuchivero, 30 km SE of Calcura/4.viii.1987, leg. S. & J. Peck/Woodland, UV light, SBP87-108 (1 ex., SEMC); ca. 20 km E Maripa, 7°26'23.2"N, 64°57'5.6"W, 45 m, flooded grassy area, 5.viii.2008, leg. Short, AS-08-074 (5 exs, MIZA, SEMC); Gran Sabana, Rio Aponwao at Highway 10, 5°50'49.2"N, 61°28'2.4"W, 1340 m, small vegetated pool, 31.vii.2008, leg. Short, AS-08-060a (2 exs., SEMC); Los Pijiguaos, outcrop/morichal, 6°35.617'N, 66°49.238'W, 80 m, algae on rocky margin of morichal, 12.i.2009, leg. Short, García, Camacho, Miller, & Joly, VZ09-0112-01B (1 ex., SEMC). **Guárico State:** Corozo Pando (8 km N.), 17–18.vi.1984, leg. F.W. Eiland & V. Linares, blacklight (8 exs., USNM); Same locality but 20–21.vi.1984, leg. F.W. Eiland & V. Linares, blacklight (2 exs., USNM).

#### Distribution.

Argentina, Bolivia, Paraguay, Uruguay, Venezuela (Anzoátegui, Bolívar, Guárico).

#### Remarks.

This species has been collected in a variety of standing or slack-water habitats. This species was previously recorded from Bolívar State (Oliva 1993).

### Berosus festivus

Berg, 1885

http://species-id.net/wiki/Berosus_festivus

Berosus festivus Berg, 1885: 221
Berosus (Berosus) vicarius Knisch, 1921b: 22. Synonymized by Oliva (1989: 101).
Berosus festivus Berg : Oliva (1989: 101).

#### Material examined

**(5).** **VENEZUELA: Barinas State:** Santa Barbara, iv.1981 light trap, leg. H. Martínez (5 ex., MACN).

#### Distribution.

Argentina, Brazil, Guyana, Uruguay, Venezuela (Barinas).

#### Remarks.

This species was first recorded from Venezuela by Oliva (1989) from Santa Barbara in Barinas State, although Oliva (1993) later omitted Venezuela because of some doubt about the identity of the Venezuelan specimens; Hansen (1999) based the distribution data of this species on the latter paper. The first author examined again the specimens from Barinas and confirmed they have all the characters of *Berosus festivus*.

### Berosus garciai

Oliva & Short sp. n.

urn:lsid:zoobank.org:act:90702F78-0B15-48A6-9639-EB4A925AB333

http://species-id.net/wiki/Berosus_garciai

Figs 6A–B , 7B , 29

#### Type material.

**Holotype** (male): “VENEZUELA: Amazonas State/ 5°30.311'N, 67°36.921'W/ nr. Campamento Canturama/ 14.i.2009; leg. Short, Camacho,/ Miller, García, & Joly; Orinoco/ floodplain pools; VZ09-0114-03B”, “[barcode]/ SM0844153/ KUNHM-ENT”, “HOLOTYPE/ BEROSUS/ garciai sp. n./ des. Oliva & Short 2010” (MIZA). **Paratypes (242):** **VENEZUELA: Amazonas State:** Culebra, N. Duida, 7-16.iv.1950, “J. Maldonado Capriles Coll.” (4 exs., USNM, SEMC); Same data as holotype (119 exs., SEMC, MIZA, MALUZ, NHMW, NMPC); ca. 7 km S. Samariapo, 5°10.900'N, 67°46.078'W, 95 m, roadside pond; 15.i.2009, leg. Short, Miller, García, Camacho & Joly, VZ09-0115-02X (2 exs., SEMC); at river near confluence of Orinoco/Sipapo rivers, rock pool, 15.i.2009, leg. García, VZ09-0115-01D (12 exs., SEMC); same locality but leg. Short, VZ09-0115-01C (1 ex., SEMC); S. Communidad Porvenir, 5°20.514'N, 67°45.315'W, 87 m, pool in culvert, 14.i.2009, leg. Short & García, VZ09-0115-03A (2 exs., SEMC); ca. 15 km S. Puerto Ayacucho, 5°30.623'N, 67°36.109'W, 110 m, rock pools et al., 14.i.2009, leg. Short, VZ09-0114-03B (30 exs., MIZA, SEMC); nr. Iboruwa, “Tobogancito”, 5°48.414'N, 67°26.313'W, 80 m, rock pool with detritus, 13.i.2009; leg. Short, VZ09-0113-02B (42 exs., MIZA, SEMC); S. Puerto Ayacucho, 5°36.250'N, 67°34.955'W, 96 m, 4.i.2006, leg. Short, HG-vapor light, AS-06-010 (7 exs., SEMC); S. Puerto Ayacucho, El Tobogan de la Selva, 5°23.207'N, 67°36.922'W, 125 m, margin of main slide, 14.i.2009, leg. Short, Miller & Joly, VZ09-0114-01A (1 ex., SEMC). **Bolívar State:** ca. 25 km E El Burro, 06°13.059'N, 67°14.467'W, 62 m, rocky side pools of morichal, 13.i.2009, leg. Miller, VZ09-0113-01A (22 exs., SEMC). Representative specimen will be deposited in NHW, NMPC, and USNM.

#### Diagnosis.

Within the *sticticus*-complex (small species without dorsal metallic luster), this species is remarkable by the strongly melanic labrum and by the absence of the pair of spots usually present on the elytral disc on the fourth interstria. It may be distinguished from the other new species with a similar coloring by the tenth elytral interstria wider than the eleventh, the weak anterior tooth of the mesoventral process, directed downwards and backwards, and by the apical notch in the fifth apparent ventrite, which bears a pair of teeth at bottom.

#### Description.

Body length: 2.2–2.8 mm (holotype: total length: 2.2 mm; humeral width: 1.05 mm). Shape depressed. Eyes rather prominent. Labrum strongly melanic, clypeus testaceous, in most specimens melanic at the base, frons melanic save at the anterolateral areas. Pronotum testaceous with a pair of small, round, weakly melanic spots behind the eyes. Scutellum testaceous. Elytra testaceous with small, strongly melanic spots; those usually placed on fifth interstria absent. Venter melanic, dark reddish to black. Maxillary palpi darkened on apical one-fourth. Femora with pubescent portion darkened, glabrous portion testaceous.

Clypeus convex in lateral aspect, sparsely and finely punctured. Frons with somewhat coarser punctures; frontal carina apparent; base of frons weakly reticulate. Pronotum very short and wide, about three times as wide as long; punctures round to polygonal, on disc about twice the size of an ommatidion, at the sides coarser, contiguous. Background micropunctate, shining. Scutellum with a few coarse punctures; ground rugulose, shining. Elytral striae fine with deeply impressed punctures about the size of those on pronotum. Interstriae moderately wide, flat except for the ninth, tenth and eleventh which are very weakly convex, step-shaped, bearing punctures much smaller than the serial ones; ground micropunctate. Elytral apices simple. No spine-like hairs.

Mesoventral process with large, weakly curved anterior tooth that points downwards and backwards; behind this the ventral margin finely serrate; posterior angle weakly raised. Metaventral process narrow; posterolateral angles produced into small rounded lamellae; posterior angle carinate, in lateral aspect convex, not more strongly raised than posterolateral ones. First ventrite carinate in anterior two-thirds, the carina thick at base, gradually narrowing backwards. No lateral depressions. Fifth ventrite with shallow apical notch; bottom of the later produced into a wide-based tooth.

Maxillary palpi short; second segment shorter than fourth, which is ensiform but very thick. Basal pubescence on two-thirds of mesofemora and three-fourths of metafemora, limit transverse. Protarsus of male with small adhesive soles on the two basal segments, which are weakly thickened; basal segment hardly longer than the following one; distal segment as long as the other three together. Claws slender, weakly arched, toothed at base.

Male genitalia: basal piece three-quarters of total length. Parameres curved towards the sternal side; apices narrow; short subapical row of hairs; sternal margin straight. Median lobe as long as parameres, nearly straight, with weakly swollen spindle-shaped apex.

#### Etymology.

This species is dedicated to Mauricio García, who has been an ardent student of aquatic Coleoptera of Venezuela for the last fifteen years, and friend and colleague of the authors.

#### Distribution.

Venezuela (Amazonas, Bolívar).

#### Remarks.

This species is known from pools and streams along the northwestern margin of the Guiana Shield (Fig. 29). In a few instances during the dry season, when habitats are becoming scarce, the species was collected by the thousands in rock pools along the Orinoco River.

### Berosus geayi

d’Orchymont, 1937

http://species-id.net/wiki/Berosus_geayi

Figs 12 , 25A–B

Berosus (Berosus) geayi d’Orchymont, 1937: 471.
Berosus geayi d’Orchymont: Oliva (1993: 224).

#### Material examined

**(105).** **VENEZUELA:** **Falcón State**: Médanos de Coro, 11°26.215'N, 69°40.112'W, 8 m, pond in dunes, 9.vii.2010, leg. Short & Shepard, VZ10-0709-03Z (4 exs., SEMC); SE Tocopero, 10.vii.2009, leg. Short et al., muddy pool in roadside ditch, VZ09-0710-03C (96 exs., MIZA, MALUZ, NHW, NMPC, USNM, SEMC); same data but pond margin, VZ10-0710-03B (1 ex., SEMC); same data but muddy pool in roadside ditch, leg. Sites, VZ10-0710-03S/L-1067 (4 exs., SEMC).

**Figure 12.**
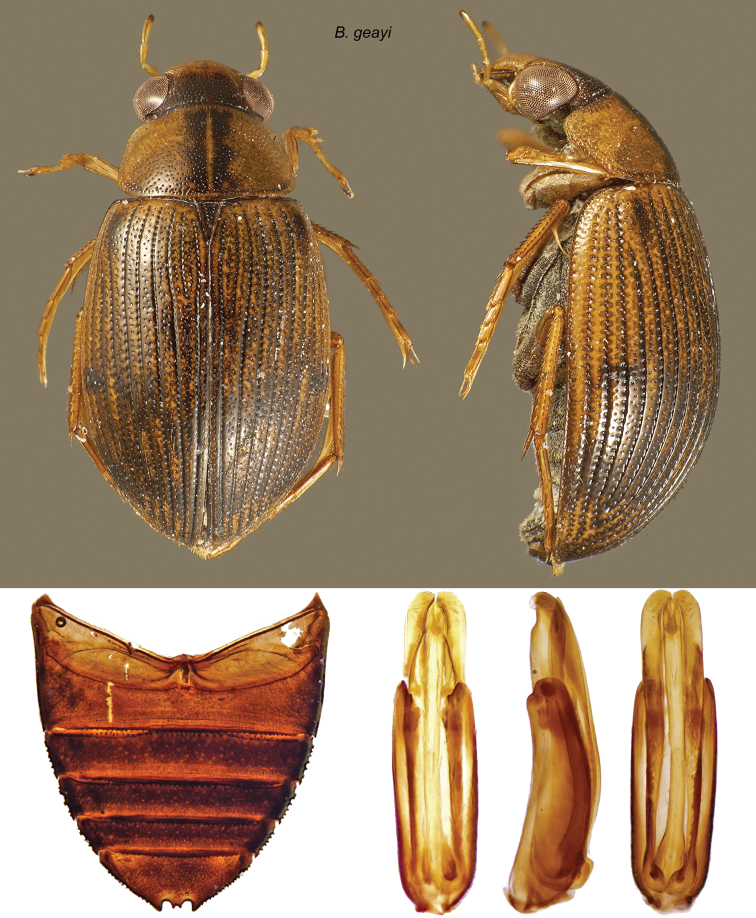
*Berosus geayi*. Dorsal and lateral habitus, abdomen, and aedeagus.

#### Distribution.

Brazil (Pernambuco), French Guiana, Paraguay, and Venezuela (Falcón).

#### Remarks.

This species is thus far known only from a few closely-situated localities near the Caribbean coast of Falcón State where it was found in ponds and a roadside ditch. Oliva (2007) records this species from northern Brazil, French Guiana and Paraguay (Central), always in small numbers, and suggests that this species might have a widespread South American distribution, like *Berosus patruelis* Berg. The findings from Venezuela suggest this is species associated with the Orinoco basin and the coastal plains between the latter and the mouth of the Amazonas, and that Paraguay may be the southern limit of its distribution

### Berosus ghanicus

d’Orchymont, 1941

http://species-id.net/wiki/Berosus_ghanicus

Berosus ghanicus d’Orchymont, 1941: 20.
Berosus ghanicus d’Orchymont: Oliva (1989: 158).

#### Material examined

**(1):** **VENEZUELA:** **Bolívar State:** Cuchivero, 30 km SE of Caicara, 4.viii.1987, leg. S. & J. Peck, Woodland, UV light, SBP87-108 (1 female, SEMC).

#### Distribution.

Brazil (Espiritu Santo), Venezuela (Bolívar).

#### Remarks.

The only known Venezuela specimen was collected at a light trap.

### Berosus guyanensis

Queney, 2006

http://species-id.net/wiki/Berosus_guyanensis

Fig. 13

Berosus guyanensis Queney, 2006: 206.

#### Material examined

**(9):** **VENEZUELA:** **Bolívar State:** Chivaton Hotel, on rd. to Kavanayen, 1370 m, 28.vi.1987, leg. M.A. Ivie, at light (1 ex., MTEC); E. of Kavanayen, 5°37'53.8"N, 61°41'12.9"W, 1330 m, small stream, 1.viii.2008, leg. Short & García, AS-08-061 (6 exs., SEMC, MIZA); 1 km S. San Francisco, 5°2.623'N, 61°6.083'W, 885 m, morichal stream/marsh, leg. Short & García, AS-08-068 (1 ex., SEMC); Gran Sabana, Rio Aponwao at Highway 10, 5°50'49.2"N, 61°28'2.4"W, 1340 m, small vegetated pool, 31.vii.2008, leg. Short, AS-08-060a (1 ex., SEMC);

**Figure 13.**
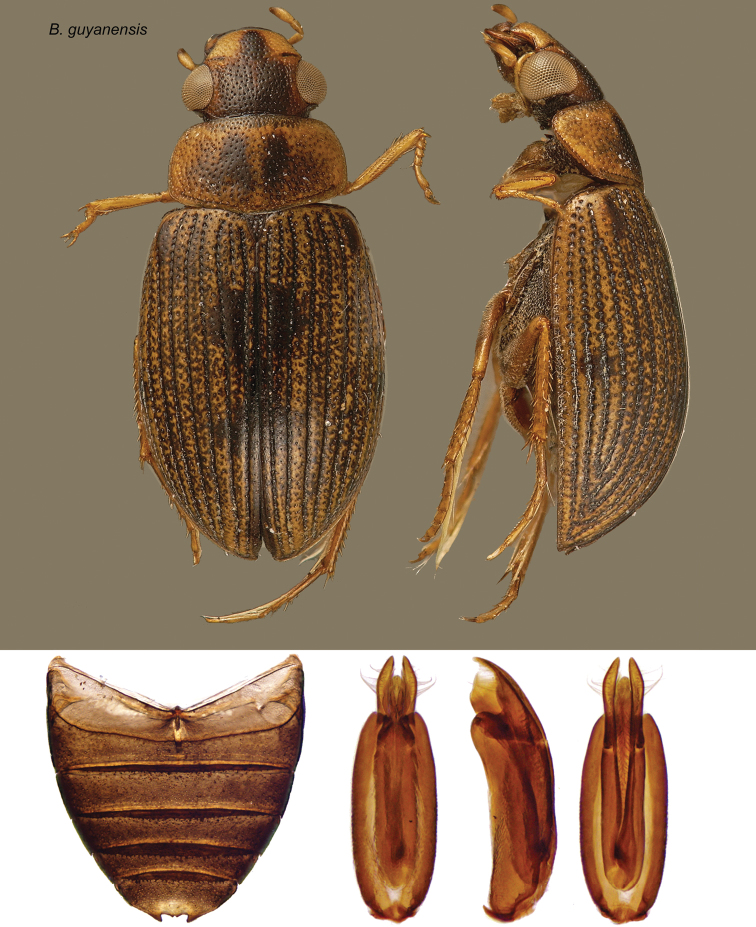
*Berosus guyanensis* Queney. Dorsal and lateral habitus, abdomen, and aedeagus.

#### Distribution.

French Guiana, Venezuela (Bolívar). New record for Venezuela.

#### Remarks.

This species was collected in small, open streams and at lights along the eastern edge of the Gran Sabana region in southern Venezuela.

### Berosus holdhausi

Knisch, 1921

http://species-id.net/wiki/Berosus_holdhausi

Figs 14C , 15B

Berosus holdhausi Knisch, 1921: 12.
Berosus holdhausi Knisch: Oliva (1989: 86).

#### Material examined 

**(14):** **VENEZUELA:** **Anzoátegui State:** Transect 1, 09°17'16.3"N, 64°13'39.1"W, 274 m, 15.viii.2009, temporary pond on side of road, leg. R. Cordero, VZ09-0815-12A (2 exs., SEMC). **Bolívar State:** ca. 40 km SE Upata, junction of Rts. 10 & 2, 7°52'1.7"N, 62°3'46.6"W, 260 m, roadside ditch, 30.vii.2008; leg. Short, AS-08-052 (12 exs., MIZA, SEMC).

**Figure 14.**
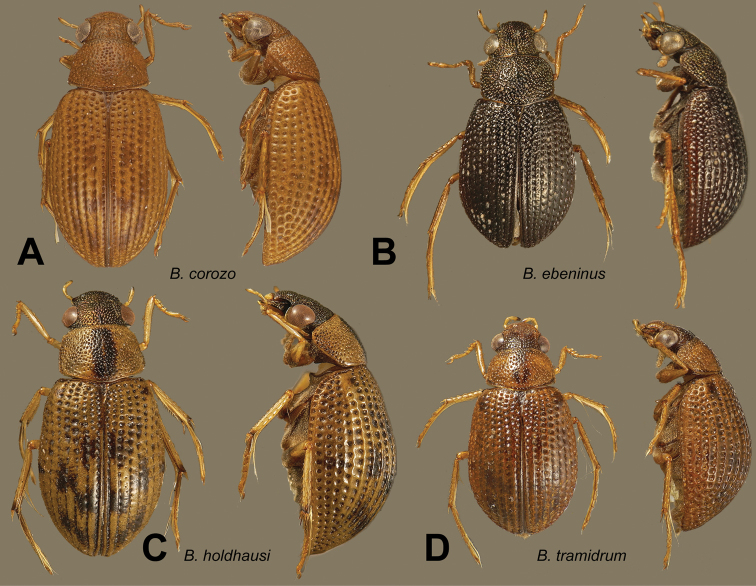
Dorsal and lateral habitus views of *Berosus* spp. **A** *Berosus corozo* sp. n. **B** *Berosus ebeninus* sp. n. **C** *Berosus holdhausi* **D** *Berosus tramidrum* sp. n.

**Figure 15.**
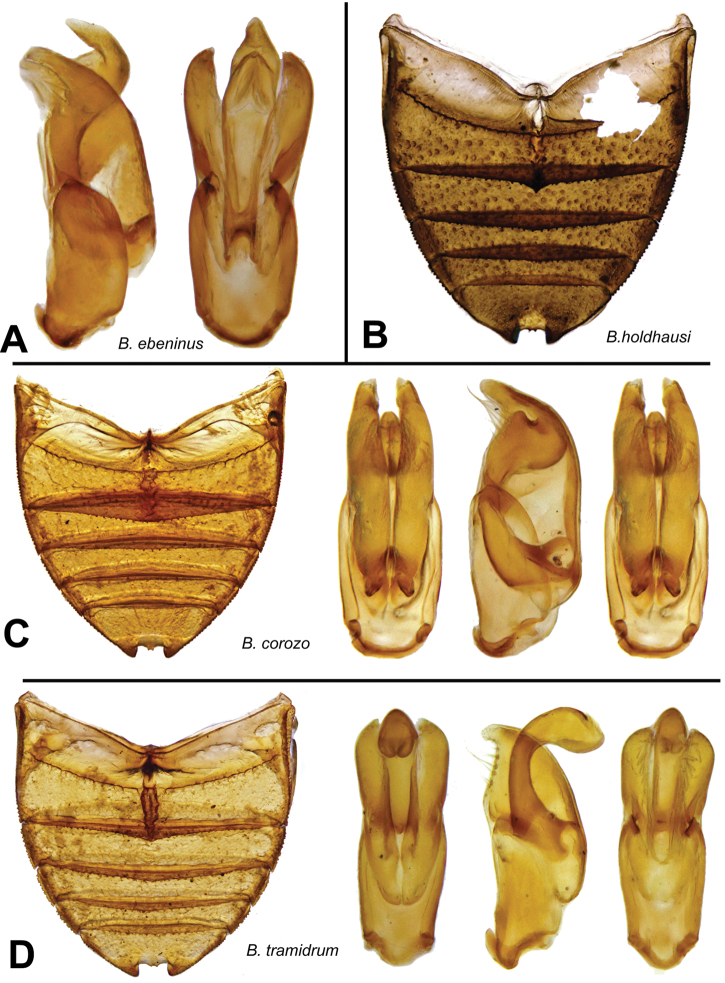
Details of *Berosus* spp. **A** *Berosus ebeninus* sp. n., aedeagus **B** *Berosus holdhausi*, abdomen **C** *Berosus corozo* sp. n., abdomen and aedeagus, **D**) *Berosus tramidrum* sp. n., abdomen and aedeagus.

#### Distribution.

Argentina, Bolivia, Brazil, Paraguay, Venezuela (Anzoátegui, Bolívar).

**Remarks.** Both collecting events for this species were in temporary standing waters.

### Berosus humeralis

Oliva & Short sp. n.

urn:lsid:zoobank.org:act:DC499216-B65A-4FBF-A425-6F0466A0EFB8

http://species-id.net/wiki/Berosus_humeralis

Figs 16A , 17A , 27

#### Type material.

**Holotype** (male): “VENEZUELA: Amazonas State/ 5°30.623'N, 67°36.109'W, 110 m/ ca. 15 km S. Puerto Ayacucho/ 14.i.2009; rock pools et al./ leg. A.Short; VZ09-0114-03B”, “[barcode]/ SM0843787/ KUNHM-ENT”, “HOLOTYPE/ BEROSUS/ humeralis sp. n./ des. Oliva & Short 2010” (MIZA). **Paratypes (12): VENEZUELA: Amazonas State:** ca. 15 km S. Puerto Ayacucho, 5°30.623'N, 67°36.109'W, 100 m, pool at base of outcrop, 14.ix.2007, leg. Short, AS-07-011a (1 ex., SEMC); same data as holotype (5 exs., SEMC, MIZA); at river near confluence of Orinoco/Sipapo rivers, rock pool, 15.i.2009, leg. García, VZ09-0115-01D (1 ex., SEMC). **Bolívar State:** Los Pijiguaos, 6°35.617'N, 66°49.238'W, 80 m, morichal/rock outcrop, 6.iii.2008, leg. Short, García, & Joly, AS-08-076 (2 exs., SEMC); same locality but 8.vii.2010, stream on side, leg. Short, Tellez, & Arias, VZ10-0708-01B (3 exs., SEMC).

#### Diagnosis.

**Figure 16.**
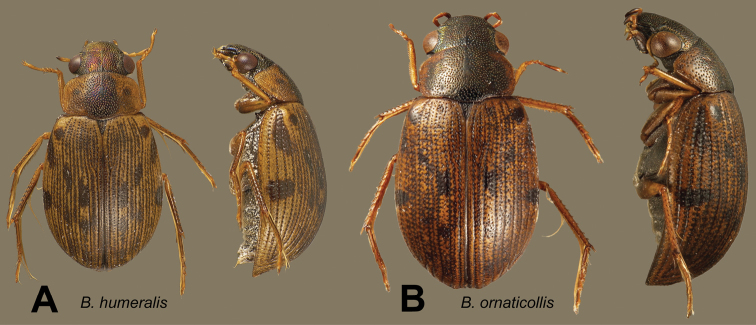
Dorsal and lateral views of *Berosus* spp. **A** *Berosus humeralis* sp. n. **B** *Berosus ornaticollis* sp. n.

**Figure 17.**
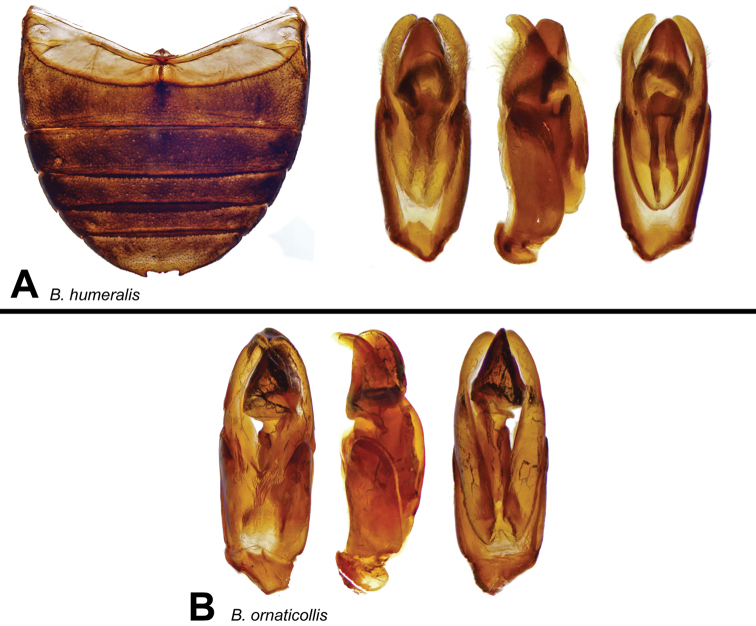
Details of *Berosus* sp. n. **A** *Berosus humeralis* sp. n., abdomen and aedeagus **B** *Berosus ornaticollis* sp. n., aedeagus.

Moderate-sized, broad-shaped, not very convex species with strong metallic luster on dorsum of head and often with weak metallic sheen on medial spot on pronotum. Elytra with small, well-defined black spots, including an additional pair below the humeral humps (Fig. 16A). Mesoventral process entirely laminar. First ventrite carinate in anterior half. Male genitalia as in Fig. 17A. The extended subhumeral spots distinguish *Berosus humeralis* from the closely related *Berosus ornaticollis* sp. n., besides the other characters listed in the key. There are hardly any species that may be confused with these two.

#### Description.

Body length: 3.7–4.0 mm. Shape short and broad, with prominent humeral humps, but rather depressed. Eyes moderately prominent in both sexes. Labrum melanic at base, testaceous on the distal margin. Dorsum of head melanic with strong metallic luster. Pronotum testaceous with a median melanic spot covering approximately two-fifths of total pronotal width, without testaceous median line, in most specimens with metallic sheen. Scutellum melanic. Elytra testaceous with small melanic spots as in *Berosus ornaticollis* sp. n., but the additional subhumeral spots more extensive, taking up the interstriae 8 and 9 and about half of 10. Venter of thorax and abdomen brown to dark brown. Maxillary palpi with apical palpomere darkened on distal quarter. Femora with pubescent portion darkened, glabrous portion testaceous.

Head densely punctured, punctures on clypeus about the size of an ommatidion, on frons slightly larger. Pronotal punctures slightly larger than ones on head, round, moderately dense, spaced by the equivalent of 2–4 times their diameter; surface sparsely and finely micropunctate, shining. Scutellum with a few deeply impressed punctures, smaller than pronotal ones; surface shining. Elytral striae fine on disc, the external ones a little more shallow, with dense punctures about the same size as the pronotal ones, not overflowing except on a short stretch of striae 6–8 on the posterior half of the elytra. Interstriae wide, flat, bearing punctures smaller than those on striae, irregularly uniseriated; outer interstriae slightly convex; background smooth. Elytral apices simple, slightly more pointed and outwardly deflexed in females; spine-like hairs absent.

Mesoventral process small, laminar, with curved anterior tooth pointing downwards, a little thickened; posterior tooth raised but less prominent than anterior one. Metaventral process as in *Berosus ornaticollis*. First ventrite carinate medially in the anterior half, carina broadened and lowered behind metacoxae; without lateral depressions. Ventrites 2–4 not carinate. Fifth ventrite with shallow apical notch, set with two straight, sharp teeth.

Maxillary palpi short, thick. Meso- and metafemora with pubescence on basal half and three-fifths respectively, limit oblique. Protarsus of male with basal tarsomeres strongly enlarged, with adhesive sole; second without sole. Claws fine, toothed.

Male genitalia (Fig. 17A): basal piece about two-fifths of total length. Parameres narrow, rounded at apex, in tergal aspect elbowed and curved inwards. Row of hairs rather long. Median lobe a little shorter than parameres, complex.

#### Etymology.

The name refers to the characteristic subhumeral spots, taking up part of the striae 8–10.

#### Distribution.

Venezuela (Amazonas, Bolívar).

#### Remarks.

This species is known only from granite outcrops in along the northwestern fringe of the Guiana Shield. It has only been found in distinctive “rock pools” that collect rainwater or in small streamlets that drain such pools (Fig. 27).

### Berosus jolyi

Oliva & Short sp. n.

urn:lsid:zoobank.org:act:3BF021DF-717C-44B8-8142-4CBE14F909CA

http://species-id.net/wiki/Berosus_jolyi

Fig. 18

#### Type Material.

**Holotype** (male): “VENEZUELA: Anzoategui State/ 9°06'42.6"N, 64°09'20.2"W, 228 m/ Transect #1; 12.viii.2009/ permanent pond/ leg. R. Cordero; VZ09-0812-06A”, “HOLOTYPE/ BEROSUS/ jolyi sp. n./ des. Oliva & Short 2010” (MIZA). **Paratypes (30): VENEZUELA: Anzoátegui State:** same data as holotype (29 exs., MIZA, MALUZ, NHMP, NHMW, SEMC);Transect 1, 09°16'34.6"N, 64°13'39.3"W, temporary pool along the road, 259 m, 24.viii.2009, leg. R. Cordero, VZ09-0814-12A (1 ex., MIZA).

#### Diagnosis.

**Figure 18.**
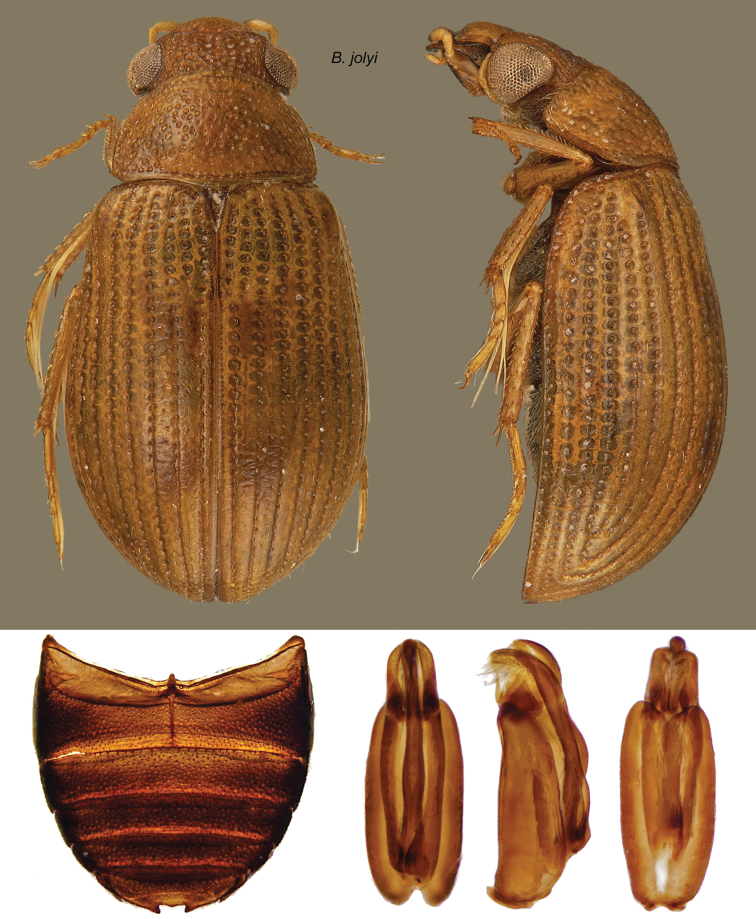
*Berosus jolyi* sp. n. Dorsal and lateral habitus, abdomen, and aedeagus.

Within the *sticticus*-complex (small species without metallic luster on dorsum), this species is remarkable by the deeply melanic labrum and the absence of the pair of elytral spots on the fourth interstriae. It may be distinguished from *Berosus garciai* sp. n., which has a similar coloring, by the tenth elytral interstria not wider than the eleventh, the strong anterior tooth of the mesoventral process, directed downwards, and by the apical notch in the fifth apparent ventrite, which bears a single tooth at bottom. The parameres of *Berosus jolyi* (Fig. 18) are more strongly curved in lateral aspect than those of *Berosus garciai* (Fig. 7B).

#### Description.

Body length: 2.3–3.1. Shape depressed. Labrum deeply melanic; clypeus testaceous; frons testaceous darkened at base. Maxillary palpi melanic on apical one-third of apical palpomere. Head and pronotum reddish in typical series; elytra testaceous with small reddish spots, the pair of spots on interstriae fourth absent. Venter blackish. Femora testaceous in typical series. Eyes of males not prominent.

Head and pronotum with moderately coarse punctures, on pronotal disc elliptical and coarser (more than twice the size of an ommatidion). Ground sparsely but deeply micropunctate. Lateral margins of pronotum entire. Elytral striae coarsely and deeply punctate, the spaces between punctures sunken with respect to interstria, so that the striae are distinctly groove-like; punctures on outer striae subquadrate. Inner interstriae about 3 times as wide as striae, flat, sparsely and finely, but deeply punctate. Outer interstriae about the same width as striae, eleventh weakly convex on middle one-third of length, elsewhere flat. Elytral apices narrowly rounded. Mesoventral process laminar; anterior tooth acute, directed downwards; posterior angle broadly rounded, as strongly raised as the anterior tooth; in between a deep semicircular notch. Metaventral process small, weakly raised, with posterolateral angles barely produced. First ventrite with a fine carina which reaches the posterior margin; fifth with a shallow apical notch bearing a single tooth at bottom; lateral margins smooth. Femoral pubescence briefly oblique, covering about two-fifths of mesofemora and half of metafemora. Protarsi of males with soles on the two basal tarsomeres, which are a little thickened.

Male genitalia: Basal piece long, more than 3 times as long as wide, taking up about three-quarters of total length. Parameres encased in basal piece for most of their length; unencased portion strongly and regularly curved towards the sternal side, bearing a short subapical row of hairs. Median lobe a little longer than parameres, subcylindrical, with apex swollen, spindle-shaped.

#### Etymology.

This species is dedicated to Dr. Luis Jose Joly T., Curator of Coleoptera at MIZA.

#### Distribution.

Venezuela (Anzoátegui).

#### Remarks.

This species has been collected in standing waters.

### Berosus llanensis

Oliva & Short sp. n.

urn:lsid:zoobank.org:act:07C94A1B-B466-4398-B7A6-99BA4BFAA141

http://species-id.net/wiki/Berosus_llanensis

Figs 19A , 20A

#### Type material.

**Holotype** (male): “VENEZUELA: Apure State/ 7°38.660'N, 69°18.004'W, 90 m/ between “La Ye” & Bruzual/ 18.i.2009; Short, Camacho, &/ Garcia; VZ-09-0118-03X: lagoon”, “[barcode]/SM0845234/ KUNHM-ENT”, “HOLOTYPE/ Berosus/ llanensis sp. n./ des. Oliva & Short 2010” (MIZA). **Paratypes (4):** **VENEZUELA:** **Apure State:** Same data as holotype (4 exs., MIZA, SEMC).

#### Diagnosis.

This species is one of the few that have the posterior angle of the metaventral process raised into a rounded lamella higher than the posterolateral angles. This character is also found in *Berosus nigrinus* Knisch, 1921 (Brazil: Mato Grosso), *Berosus sticticus* Boheman, 1859 (Brazil: Mato Grosso, Rio de Janeiro) and *Berosus guyanensis* Queney, 2006 (French Guiana, Venezuela: Bolívar). The basal piece is longer than in *Berosus sticticus* and shorter than in *Berosus guyanensis*.

#### Description.

Body length: 2.3–2.6. Shape depressed. Eyes not prominent. Labrum weakly melanic. Clypeus testaceous with small dark triangle on the middle of the posterior margin, frons testaceous with melanic median area. Pronotum testaceous with small discal melanic spot. Scutellum weakly darkened. Elytra with small melanic spots, the pair on the fourth interstria absent in observed material. Venter weakly melanic. Maxillary palpi with apical palpomere darkened on apex. Femora with pubescent portion darkened, glabrous portion testaceous.

Punctures on clypeus round, sparse, on frons a little coarser, about twice the size of ommatidia. Pronotal punctures elliptical, moderately dense. Ground distinctly micropunctate, shining. Scutellum densely punctate. Elytral striae fine on disc, bearing punctures about the same size as those on pronotum; outer striae shallow. Interstriae flat, even the outer ones; odd-numbered ones with distinct punctures, even-shaped ones with fine or obsolete punctures. Lateral margin of elytra with a row of round punctures spaced by about twice their diameter. Elytral apices separately rounded. Spine-like hairs absent.

Mesoventral process with a large straight anterior tooth, directed downwards. Metaventral process small, with posterolateral angles produced into small triangular laminae, posterior angle raised into a rounded lamina, in lateral aspect convex, scarcely higher than posterolateral angles. First ventrite carinate, without lateral depressions. Fifth ventrite with apical notch shallow, bottom of notch weakly produced; the males bear in front of the notch two raised, sharp teeth, one behind the other.

Maxillary palpi short, thick. Basal pubescence on two-thirds of mesofemora and three-quarters of metafemora, limit transverse-oblique. Protarsus of male short, sturdy, with fourth tarsomere as long as the first three together; first tarsomere about one and a half times as long as the second, both weakly swollen. Claws weakly arched.

Male genitalia: basal piece about twice as long as wide, two-thirds of total length. Parameres curved towards sternal side, with narrowed apices, bearing a short subapical row of hairs. Sternal margin strongly convex. Median lobe a little shorter than parameres, moderately thick, curved, apex blunt.

#### Etymology.

The name alludes to the fact that this species is found on the vast plains region of central Venezuela, Los Llanos.

**Figure 19.**
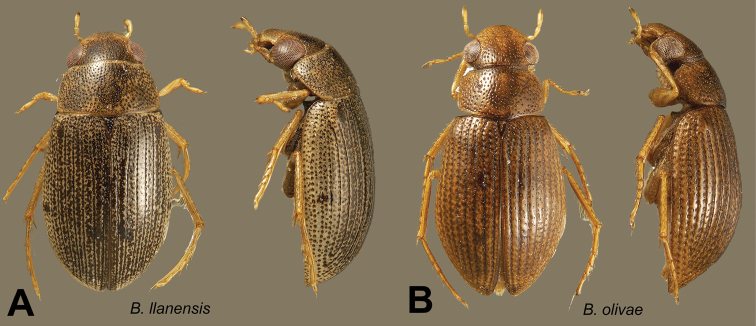
Dorsal and lateral habitus views of *Berosus* spp. **A** *Berosus llanensis* sp. n. **B** *Berosus olivae*.

**Figure 20.**
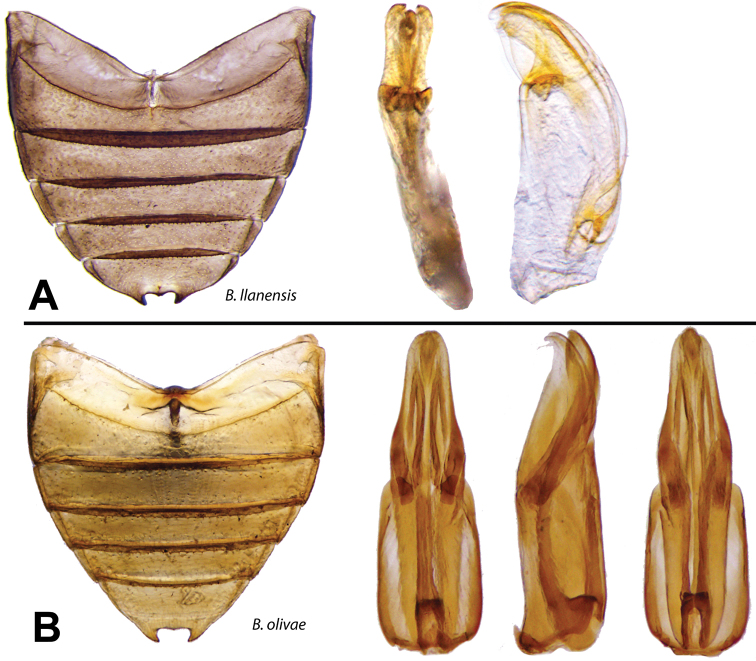
Details of *Berosus* spp. **A** *Berosus llanensis* sp. n., abdomen and aedeagus **B** *Berosus olivae*, abdomen and aedeagus.

#### Distribution.

Venezuela (Apure).

**Remarks.** This species was collected in an open marsh. The type series is partly teneral.

### Berosus marquardti

Knisch, 1921

http://species-id.net/wiki/Berosus_marquardti

Berosus marquardti Knisch, 1921: 12.
Berosus marquardti Knisch: Oliva (1989: 88).

#### Material examined.

**VENEZUELA: Apure State:** Achaguas, Samán de Apure, 25–26.vii.1997, leg. M. García (1 ex., MIZA).

#### Distribution.

Brazil,Venezuela (Apure).

### Berosus megaphallus

Oliva & Short sp. n.

urn:lsid:zoobank.org:act:45E34F19-02D3-43E3-9428-B42B505235F5

http://species-id.net/wiki/Berosus_megaphallus

Fig. 21B

#### Type material.

**Holotype** (male): “GUYANA, Karanambo/ 3°45.1'N, 59°18.6'W/ 2Apr1994, Buffalo Pond/ PJSpangler, colln #11”, “HOLOTYPE/ BEROSUS/ castaneus sp. n./ des. Oliva & Short 2010” (USNM). **Paratypes (97): GUYANA:** Karanambo, 3°45.1'N, 59°18.6'W, 31.iii.1994, ‘on bank of Rupununi’, blacklight, leg. P.J. Spangler, Collection #2 (11 exs., USNM); same locality but 2.iv.1994, ‘buffalo pond’, collection #11 (31 exs., USNM, SEMC); same locality but 2.iv.1994, Simoni Lake, collection #10 (1 ex., USNM); same locality but 2.iv.1994, Rupununi River, collection #8 (1 ex., USNM); same data but 2.iv.1994, pool in sandbar of Rupununi River, collection #9 (5 exs., USNM). **VENEZUELA: Apure State:** Fundo Mata Palito, Laguna Larga, 6°59'N, 67°17'W, 26.i.1999, leg. E. Osuna (5 exs., MIZA, SEMC). **Bolívar State:** Cuchivero, 30 km SE Caicura, 4.viii.1987, leg. S. & J. Peck, woodland UV light, SBP87-108 (1 ex., SEMC); 4°37.362'N, 61°5.679'W, 876 m,Gran Sabana, N. Santa Elena, Rio Guara at Rt. 10, 17.vii.2010, leg. Short, Tellez, & Arias, marshy area, VZ10-0717-02A (9 exs., MIZA, MALUZ, SEMC). **Delta Amacuro State**: Transect 2, Agua Negra Farm, 08°32'48.1"N, 62°09'29.6"W, 15 m, Seasonal pond, 17.viii.2009, leg. R. Cordero, VZ09-817-17A, (4 exs., SEMC); Transect 2, Agua Negra Farm, 08°32'47.5"N, 62°09'37.11"W, 15 m, Seasonal pond, 17.viii.2009, leg. R. Cordero, VZ09-817-17B, (16 exs., MIZA, MALUZ, SEMC); Transect 2, Agua Negra Farm, 08°32'47.5"N, 62°09'39.4"W, 15 m, Seasonal pond, 21.viii.2009, leg. R. Cordero, VZ09-821-17C, (6 exs., MIZA, SEMC); Transect 2, Agua Negra Farm, 08°32'47.5"N, 62°09'38.9"W, 15 m, Seasonal pond, 21.viii.2009, leg. R. Cordero, VZ09-821-17D, (5 exs., SEMC). **Monagas State:** Uverito, 24.v.1990, “En la luz”, leg. C.J. Rosales (2 exs., MIZA).

#### Diagnosis.

**Figure 21.**
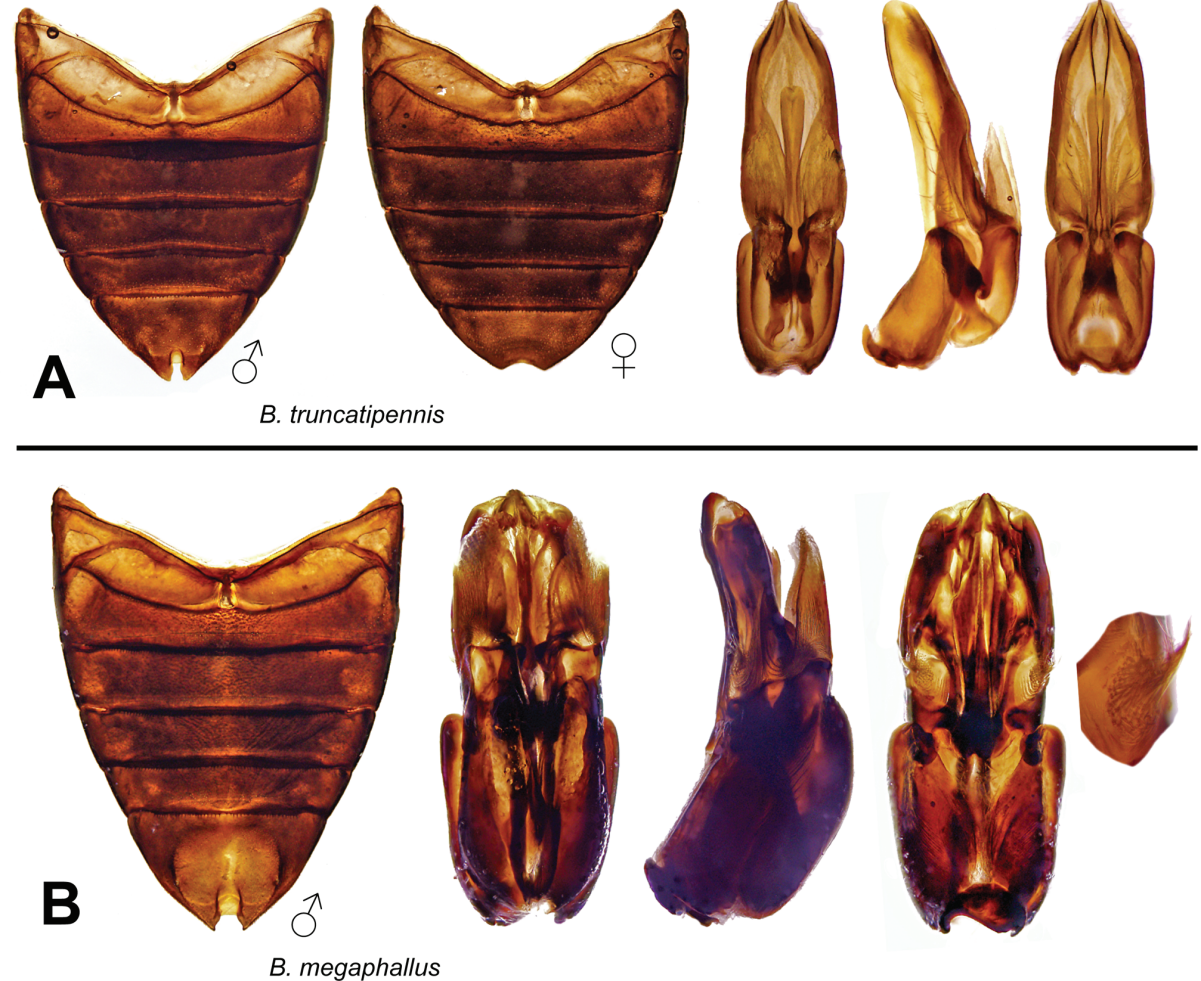
Details of *Berosus* spp. **A** *Berosus truncatipennis* abdomens (male and female) and aedeagus **B** *Berosus megaphallus* sp. n., abdomen (male) and aedeagus (including enlargement showing cluster of hairs on paramere).

Large, elongate, depressed species. Resembling large individuals of *Berosus truncatipennis* but with micropunctation finer (micropunctures about one-quarter of punctures in *Berosus truncatipennis*, one-sixth in the new species) and with sutural angle of elytra in males broadly rounded, not bearing spines. Male genitalia much larger with respect to total body size in the new species; parameres bearing a subapical tuft of hairs instead of a row; membranous appendices a little longer than the median lobe (Fig. 21B).

#### Description.

Body length: 6.0–7.7 mm. Shape elongate, depressed, not very narrow. Eyes prominent, a little more in males. Maxillary palpi slender, rather long, a little more so in males because the apical segment is longer and more slender than in females. Dorsum of head, pronotum, scutellum, and elytra testaceous, except slightly darkened on base of frons, a pair of narrow paramedial bands on pronotal disc, and small elytral spots in the usual pattern. Venter of thorax and abdomen dark brown; maxillary palpi darkened at tip of apical segment; femora with pubescent portion darkened, glabrous portion testaceous.

Dorsal sculpture moderate to fine, regular; punctures round, only on the base of frons a little elliptical. Head and pronotum with punctures about twice the size of an ommatidion; micropunctures about one-sixth the size of punctures. Elytral striae deep, well-impressed, finely punctured. Interstriae flat, wide, with moderate-sized punctures disposed in several series, on posterior half of elytra a little coarser and bearing spine-like hairs. Elytral micropunctures fine, on posterior half obsolete. Elytral apices emarginate, with acute parasutural spine; in males sutural angle broadly rounded, comprising the three inner striae; in females the emargination a little broader and the sutural angle produced into a short straight spine. Mesoventral process laminar, with large, curved, but not acute, anterior tooth, behind the margin obliquely lowered. Metaventral process narrow, with small median depression; posterolateral angles produced into triangular laminae, posterior angle not raised. First ventrite carinate medially only between metacoxae, with deep, moderately large lateral depressions. Fifth ventrite with shallow apical notch in females, in males raised into a disc around a narrow, very deep notch (Fig. 21B). Lateral margins of abdominal ventrites smooth. Meso- and metafemora with pubescence on basal three-fifths and three-quarters respectively. Male protarsi with the two basal tarsomeres thickened, bearing adhesive soles; first about twice as long as second, fourth slender, longer than 3 basal segments taken together. All claws long, slender, very weakly curved.

Male genitalia: Basal piece about two-fifths of total length. Parameres complex, in lateral aspect with wide blunt apices, with membranous appendices in a ventral position. Median lobe much shorter than parameres, a little shorter than the appendices (Fig. 21B).

#### Etymology.

Named after the comparatively large aedeagus.

#### Distribution.

Guyana andVenezuela (Apure, Bolívar, Delta Amacuro, Monagas).

#### Remarks.

All known collecting events for *Berosus megaphallus* represent standing waters, both permanent and temporary. Oliva (1989) probably handled this species (from Venezuela [Barinas] and Nicaragua [Zelaya]), but believed at the time that there was a gradient in the size of male genitalia of *Berosus truncatipennis* Castelnau, “with shorter, thicker organs in specimens of the Antilles area and Orinoco basin, and longer, more slender organs in those of the middle and lower Paraná.”

### Berosus olivae

Queney, 2006

http://species-id.net/wiki/Berosus_olivae

Figs 19B , 20B

Berosus olivae Queney, 2006: 459.

#### Material examined

**(21).** **VENEZUELA: Bolívar State:** 5°37'53.8"N, 61°41'12.8"W, 1330 m, E of Kavanayen, 1.viii.2008, 1330 m, leg. Short & García, small stream, AS-08-061 (1 ex., SEMC); 5°44'28.7"N, 61°30'54.3"W, 1290 m, E. of Kavanayen, 1.viii.2008, leg. Short & García, large vegetated marsh, AS-08-063 (20 exs., MIZA, MALUZ, SEMC, USNM);

#### Distribution.

French Guiana, Venezuela (Bolívar).

#### Remarks.

Venezuelan records for this species were collected from a large but shallow vegetated marsh and a nearby small ephemeral stream in the Gran Sabana.

### Berosus ornaticollis

Oliva & Short sp. n.

urn:lsid:zoobank.org:act:12F5D407-4167-450C-A8EC-0825685FF98C

http://species-id.net/wiki/Berosus_ornaticollis

Figs 16B , 17B , 27

#### Type material.

**Holotype** (male): “VENEZUELA: Amazonas State/ 5°30.623'N, 67°36.109'W; 100 m/ ca. 15 km S. Puerto Ayacuho/ rock pools on top [of granite outcrop]; 14.ix.2007/ AS-07-011b; leg. A.E.Z. Short”, “[barcode]/ SM0828174/ KUNHM-ENT”, “HOLOTYPE/ BEROSUS/ ornaticollis sp. n./ des. Oliva & Short” (MIZA). **Paratypes (61): VENEZUELA: Amazonas State:** nr. Iboruwa, “Tobogancito”, 5°48.414'N, 67°26.313'W, 80 m, rock pool with detritus, 7.viii.2008; leg. Short, García, & Joly, AS-08-078 (11 exs., SEMC, MIZA); Same data as holotype (7 exs., SEMC); same locality and data but 8.viii.2008, leg. Short & García, AS-08-81b (40 exs., MIZA, MALUZ, SEMC); same locality and data but 14.i.2009, VZ09-0114-03B (1 ex., SEMC). **Bolívar State:** Los Pijiguaos, 6°35.617'N, 66°49.238'W, 80 m, morichal/rock outcrop, 16.ix.2007, leg. Short, García, & Joly, AS-07-015 (1 ex., SEMC); same locality and data but 6.iii.2008, AS-08-076 (1 ex., SEMC).

#### Diagnosis.

Moderate-sized, broad-shaped, not very convex species with strong metallic luster on dorsum of head and often with weak metallic sheen on medial spot on pronotum. Elytra with small, well-defined black spots, including an additional pair below the humeral humps (Fig. 16B). Mesoventral process entirely laminar. First ventrite carinate in anterior half. Male genitalia as in Fig. 17B. The melanic spots below the humeral humps are most distinctive; they are shared with *Berosus humeralis* sp. n., but they are less extended, taking up only a part of the ninth interstria. This species belongs to the *auriceps*-complex. The shape of the median lobe is most characteristic; in *Berosus auriceps* the median lobe is narrowly rounded at the apex; in *Berosus aulus* d’Orchymont, 1941 the moderately thick median lobe is much shorter than the parameres, and in *Berosus ethmonotus* Oliva, 1989 the apices of the parameres are broader and directed outwards. The new species may be distinguished from all the others by the toothed claws.

#### Description.

Body length 3.4–4.6 mm. Shape short and wide, moderately convex. Eyes moderately prominent in both sexes. Labrum black, dorsum of head melanic with strong metallic luster. Pronotum with remarkably large central melanic spot, without a testaceous median line, in most specimens with a metallic sheen; sides of the pronotum testaceous. Scutellum black. Elytra testaceous with small melanic spot in the normal generic pattern, save for a pair of additional spots which delimit the humeral humps on their ventral side and extend over a short stretch of the ninth interstria. Venter of thorax and abdomen melanic. Maxillary palpi darkened on apical one-third of distal segment. Femora with pubescent portion darkened, glabrous portion testaceous.

Head coarsely and densely punctured, punctures on frons about twice the size of ommatidia. Pronotal punctures larger than the ones on head, round, dense, irregular in spacing. Ground sparsely and finely micropunctate, shining. Scutellum with a few deeply impressed punctures, smaller than pronotal ones; ground alutaceous. Elytral striae shallow, not distinctly impressed, with coarse punctures about the same size as pronotal ones, overflowing outwards. Interstriae wide, flattened, bearing punctures smaller than those on striae, 1–2 seriated; outer interstriae slightly convex; background smooth in males, reticulate in females. Elytral apices simple. Spine-like hairs absent.

Mesoventral process small, laminar, with curved anterior tooth pointing downwards, a little thickened; posterior tooth raised but less prominent than anterior one. Metaventral process wide, flat. First ventrite carinate in anterior half, without lateral depressions. Ventrites second to fourth not carinate. Fifth ventrite with shallow apical notch, the bottom of the latter bearing two short rounded teeth.

Maxillary palpi short, with second palpomere short and thick. Basal pubescence of mesofemora and of metafemora slightly oblique, extending to a little less than half of the femoral length on anterior margin, and a little more than half on the posterior margin. Protarsus of male with basal tarsomere weakly thickened, bearing adhesive sole, about twice as long as the second tarsomere that does not bear a sole. Claws weakly arched, angular at base, bearing a small sub-basal tooth.

Male genitalia (Fig. 17C): basal piece two-fifths of total length. Parameres acuminate, in lateral aspect almost sickle-shaped, in tergal aspect weakly and regularly curved inwards. Apices broadly rounded; short subapical row of hairs. Median lobe a little shorter than the parameres, cylindrical and rather slender in basal three-fifths, very thick and subconical in apical two-fifths, with a sternal ridge.

#### Etymology.

The name alludes to the deeply sculptured, shining, brightly colored pronotum of this species.

#### Distribution.

Venezuela (Amazonas, Bolívar).

#### Remarks.

As with *Berosus humeralis* sp. n., this taxon is known only from granite outcrops in along the northwestern fringe of the Guiana Shield. It has only been found in distinctive “rock pools” that collect rainwater or in small streamlets that drain such pools. The allied *Berosus auriceps* and *Berosus aulus* are found in marginal pools of streams with rocky to sandy substrates (Oliva 1989).

### Berosus pallipes

Brullé, 1841

http://species-id.net/wiki/Berosus_pallipes

Berosus pallipes Brullé, 1841: 59.
Berosus pallipes Brullé: Oliva (1989 : 143).
Berosus engelharti Jensen-Haarup, 1906: 50. Synonymized by Knisch (1924: 272).
Berosus licayensis Moroni, 1970: 3. Synonymized by Moroni (1985: 169).

#### Material examined.

None.

#### Distribution.

Argentina, Brazil, Chile, Uruguay, Venezuela (Barinas).

#### Remarks.

Originally described from Argentina, it was recorded from Venezuela (Barinas: Santa Barbara) by Oliva (1989).

### Berosus patruelis

Berg, 1885

http://species-id.net/wiki/Berosus_patruelis

Figs 22A , 23A

Berosus patruelis Berg, 1885: 220.
Berosus patruelis : Oliva (1989: 122).
Berosus carinatus Mouchamps, 1963: 134. Synonymized by Oliva (1989: 122).

#### Material examined

**(424).** **VENEZUELA:** **Anzoátegui State:** Transect 1, permanent pond with grass, 12.viii.2009, leg. Cordero, VZ09-0812-02A (9 exs., SEMC); 09°17'16.3"N, 64°13'39.1"W, 274 m, Temporal pond aside the road, 15.viii.2009, leg. Cordero, VZ09-815-12A (1 ex.). **Apure State:** ca. 18 km S. San Fernando, 7°33.869'N, 67°38.456'W, 50 m, marsh along road; 4.i.2006, leg. Short, AS-06-008 (13 exs.); 44 km N. Rio Capanaparo, 7°20.175'N, 67°43.868'W; 49 m, marsh at sand dunes, 11.ix.2007, leg. Short, AS-07-004 (2 ex.); on side road ca. 10 km E. Mantecal, 7°37.298'N, 69°3.679'W, 83 m, marshy area and pool by road, 18.i.2009, leg. Short, García, & Camacho, VZ09-0118-02X (8 exs.); between “La Ye” & Bruzual, 7°38.660'N, 69°18.004'W, 90 m, lagoon, 18.i.2009, Short, Camacho & García, VZ09-0118-03X (34 exs.). **Aragua State:** “Venez. Cagua/Edo. Aragua/28-VI-1961 Bordon.” (6 exs., MSUC). Road between Rio Capanaparo and Rio Cinaruco; Medanos de la Soledad, 7°20.175'N, 67°43.868'W, 49 m, marshy area, 17.i.2009, leg. Short, Camacho, Miller, VZ09-0117-02X (45 exs.); Rt. 19, E. Apurito, 7°54.823'N, 68°25.782'W, shaded pond by road, 18.i.2009, leg. Short, García, & Camacho, VZ09-0118-01X (7 exs.). **Barinas State:** 10 km NE Barinas, 12.ii.1969, P. & P. Spangler (2 exs., USNM); Small stream, c. 20 km S. Ciudad Bolivia, stream margins, 25.i.2012, leg. Short, Arias, Gustafson, VZ12-0125-01A (18 exs.); Marsh on large Hacienda, ca. 13 km SE of Ciudad Bolivia, , Marsh, 25.i.2012, leg. Short, Arias, Gustafson, VZ12-0125-02A (80 exs.); River E. of Santa Barbara, big sidepool of river, 26.i.2012, leg. Short, Arias, Gustafson, VZ12-0126-01B (5 exs.). **Cojedes State:** Rio Caiman Grande at San Brano, c. 7.5 km NW of El Pao, stream margins, 20.i.2012, leg. Short, Arias, Gustafson, VZ12-0120-03A (2 exs., SEMC); Embalsa El Pao next to main church, marsh margins, 21.i.2012, leg. Short, Arias, Gustafson, VZ12-0121-01A (1 ex.); c. 2 km S. La Galera, between El Pao and El Baul, Marsh/pond, 21.i.2012, leg. Short, Arias, Gustafson, VZ12-0121-02A (3 exs.). **Falcón State:** E. Dabajuro, river at bridge crossing on Rt. 3, 11°02.305'N, 70°38.467'W, 98 m, Gravel and sand margins of river and associated side pools, 8.vii.2009, Short, Gustafson, & Inciarte, VZ09-0708-01A (1 ex.); SE of Tocopero, 11°26.922'N, 69°13.109'W, 12 m, Puddle in roadside ditch and culvert, 10.vii.2009, Short, Gustafson, Camacho,, VZ09-0710-03C (45 exs.); SE of Tocopero, 11°26.922'N, 69°13.109'W, 12 m, Puddle in roadside ditch and culvert, 10.vii.2009, leg. Sites, VZ09-0710-03S (8 exs.). **Guárico State:** Corozo Pando (8 km N.), 11.vi.1984, leg. F.W. Eiland (2 exs., USNM); Same locality but 17-18.vi.1984, leg. F.W. Eiland & V. Linares, blacklight (30 exs., USNM); Same locality but 20–21.vi.1984, leg. F.W. Eiland & V. Linares, blacklight (1 ex., USNM); 15 km S. Calabozo, 9–13.ii.1969, Lago de los Patos, P. & P. Spangler (5 exs., USNM); 20 km S. Calabozo, 8–13.ii.1969, Rio Orituco, P&PS (1 ex., USNM); Calabozo (44 km S.) Hato Masaguaral, 6.iii.1986, coln #31, P. Spangler & S. Beaujon (2 exs., USNM); nr. Socorro, 8°59'1.0"N, 65°44'18.8"W, 100 m, muddy ditch, 29.vii.2008, leg. Short & García, AS-08-050 (1 ex.); W. of Las Mercedes, 9°5.067'N, 66°28.500'W, 153 m, 8.i.2009, leg. Short & Miller, roadside pond, VZ09-0108-04A (2 exs.); ca. 65 km S. Las Mercedes, 8°31.705'N, 66°22.602'W, 145 m, large lagoon with vegetation, 9.i.2009, leg. Short, Miller, García, & Camacho, VZ09-0109-01X (10 exs.); same locality but 5.vii.2010, leg. Short, Tellez & Camacho, VZ10-0705-01A (1 ex.); same locality but 6.vii.2010, leg. Short, Tellez, Camacho, & Arias, VZ10-0706-03B, (18 exs.); San Nicolasito Field Station, 8°8.677'N, 66°24.263'W, 50 m, pool without fish, 10.i.2009, leg. Miller & García, VZ09-0110-03A (1 ex.). **Tachira State:** Mata de Limon, small lagoon on finca, marsh, 26.i.2012, leg. Short, Arias, Gustafson, VZ12-0126-02A (24 exs.). **Trujillo State:** c. 10 km E. La Cieba, by Cemetary, Lagoon, 28.i.2012, leg. Short, Arias, Gustafson, VZ12-0128-06A (8 exs.); Nr. Agua Viva, Small creek, 29.i.2012, leg. Short, Gustafson, VZ12-0129-01A (3 exs.). **Zulia State:** Puente del Zulia, muddy pools in pasture, 27.i.2012, leg. Short, Arias, Gustafson, VZ12-0127-01B (13 exs.); E. San Carlos Del Zulia, small lagoon, 27.i.2012, leg. Short, Arias, Gustafson, VZ12-0127-02A (1 ex.); Sabana de Machango, artificial lagoon, 29.i.2012, leg. Short, Arias, Gustafson, VZ12-0129-03A (11 exs.).When not indicated, material is distributed between MIZA, MALUZ, SEMC, and NMW.

**Figure 22.**
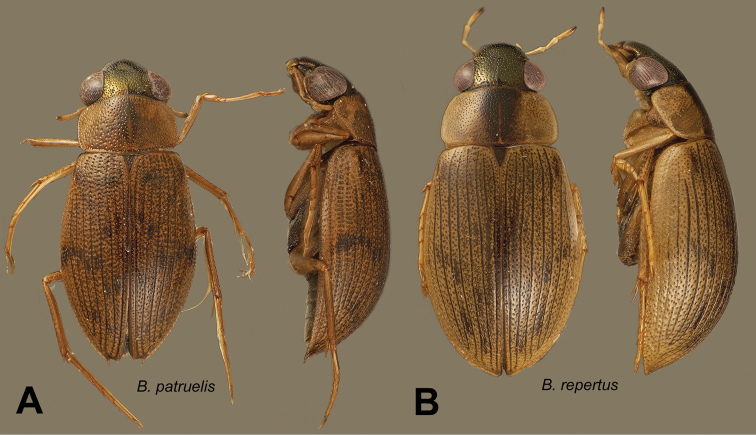
Dorsal and lateral habitus of *Berosus* spp. **A** *Berosus patruelis* **B** *Berosus repertus* sp. n.

**Figure 23.**
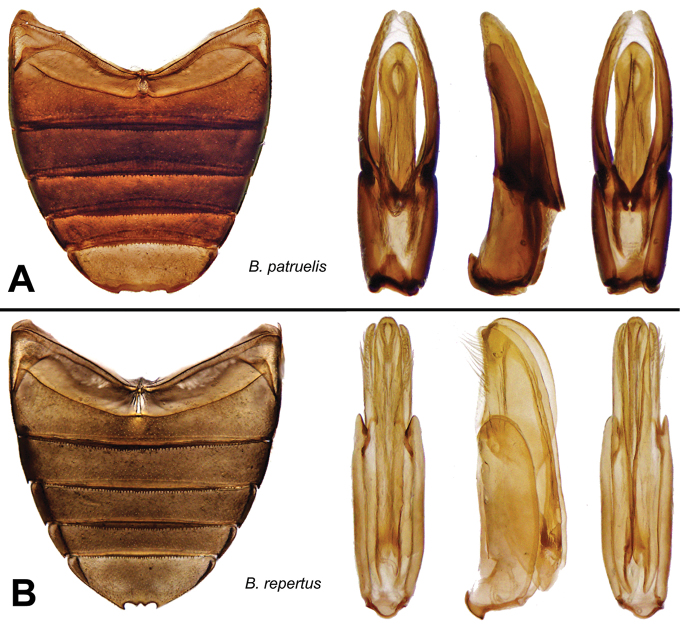
Details of *Berosus* spp. **A** *Berosus patruelis*, abdomen and aedeagus **B** *Berosus repertus* sp. n., abdomen and aedeagus.

#### Distribution.

Widespread in South America, including Argentina, Bolivia, Brazil, Paraguay, Venezuela (Anzoátegui, Apure, Aragua, Barinas, Cojedes, Falcón, Guárico, Tachira, Trujillo, Zulia). Previously recorded in Apure State (Oliva 2002).

#### Remarks.

This very common species is mostly encountered in standing waters such as marshes and roadside ditches. It has not yet been encountered south of the Orinoco in Venezuela.

### Berosus pluripunctatus

Mouchamps, 1963

http://species-id.net/wiki/Berosus_pluripunctatus

Berosus (Berosus) pluripunctatus Mouchamps, 1963: 137.
Berosus pluripunctatus Mouchamps: Oliva (1989: 151).

#### Material examined.

None.

#### Distribution.

Brazil (Mato Grosso), Venezuela.

#### Remarks.

Originally described from Brazil (Mato Grosso), Oliva (1993) subsequently recorded it from Venezuela (Barinas State: Santa Barbara).

### Berosus reticulatus

Knisch, 1921

http://species-id.net/wiki/Berosus_reticulatus

Figs 2A , 4B

Berosus (Enoplurus) reticulatus Knisch, 1921: 62.
Berosus reticulates Knisch: Oliva (1989: 155).

#### Material examined

**(161).** **VENEZUELA: Anzoátegui State**: 9°06'42.6"N, 64°09'20.2"W, 228 m, Transect #1, 12.viii.2009, permanent pond, leg. R. Cordero, VZ09-0812-06A (1 ex., SEMC). **Barinas State:** 10 km NE Barinas, 12.ii.1969, leg. P. & P. Spangler (1 ex., USNM). **Bolívar State:** Cuchivero, 30 km SE of Caicara, 4.viii.1987, leg. S. & J. Peck, Woodland, UV light, SBP87-108 (2 exs., SEMC); ca. 40 km SE Upata, junction of Rts. 10 & 2, 7°52’1.7"N, 62°3'46.6"W, 260 m, roadside ditch, 30.vii.2008, leg A. Short, AS-08-052 (1 ex., SEMC); nr. Rio Sakaika, 5°34'29.8"N, 61°18'43.4"W, 1100 m; roadside pond, 2.viii.2008, leg. Short & García AS-08-067 (1 ex., SEMC). **Falcón State:** ca. 18 km E. Urumaco, Lagoon along Rt. 3, W. of Coro, 11°14.228'N, 70°05.312'W, 79 m, marginal, vegetated areas of lagoon, 8.vii.2009, leg. Short, Gustafson, García, Camacho, & Inciarte, VZ09-0708-02A (3 exs., SEMC, MIZA); Médanos de Coro, pond in dunes, 11°26.215'N, 69°40.112'W, 8 m, pond in dunes, 9.vii.2009, leg. Shepard & Sites, VZ09-0709-03D (1 ex., SEMC). **Guárico State:** Corozo Pando (8 km N.), 11.vi.1984, leg. F.W. Eiland (3 exs., USNM); same locality but 17–18.vi.1984, leg. F.W. Eiland & V. Linares, blacklight (138 exs., USNM), same locality but, 20-21.vi.1984, leg. F.W. Eiland & V. Linares, blacklight (9 exs., USNM); 15 km S. Calabozo, 9-13.ii.1969, Lago de los Patos, leg. P. & P. Spangler (1 ex., USNM).

#### Distribution.

Widespread in South America including Venezuela (Anzoátegui, Barinas, Bolívar, Falcón, Guárico).

#### Remarks.

All known collecting events in Venezuela are from standing waters (ponds, lagoons, ditches) or light traps near such habitats.

### Berosus ruffinus

d’Orchymont, 1946

http://species-id.net/wiki/Berosus_ruffinus

Figs 3B , 24 , 29

Berosus ruffinus d’Orchymont, 1946: 1.
Berosus ruffinus d’Orchymont: Oliva (1989: 173).

#### Material examined

**(24):** **VENEZUELA:** **Amazonas State:** nr. Campamento Canturama, 5°30.311'N, 67°36.921'W, Orinoco flood plain pools, 14.i.2009, leg. Short, Camacho, Miller, García & Joly, VZ09-0114-02A (24 exs., MIZA, MALUZ, SEMC, NHW, NMPC, USNM).

**Figure 24.**
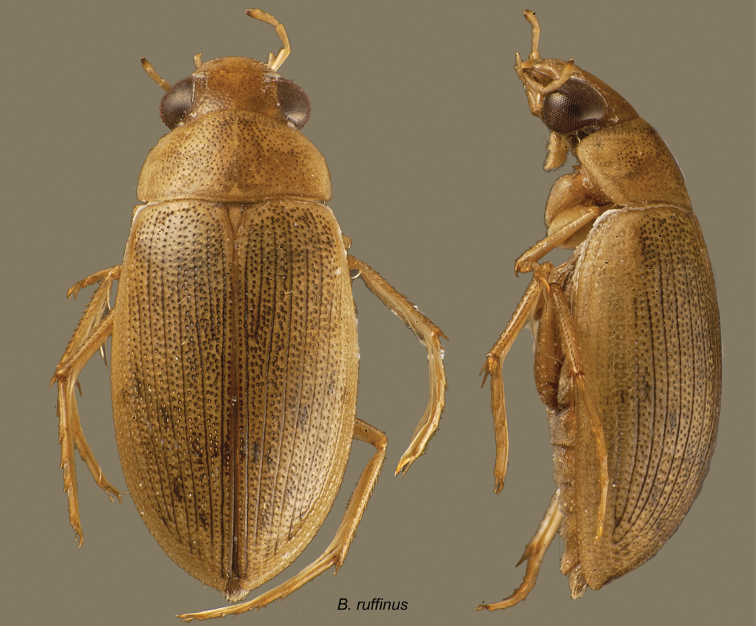
*Berosus ruffinus*, dorsal and lateral habitus.

**Figure 25.**
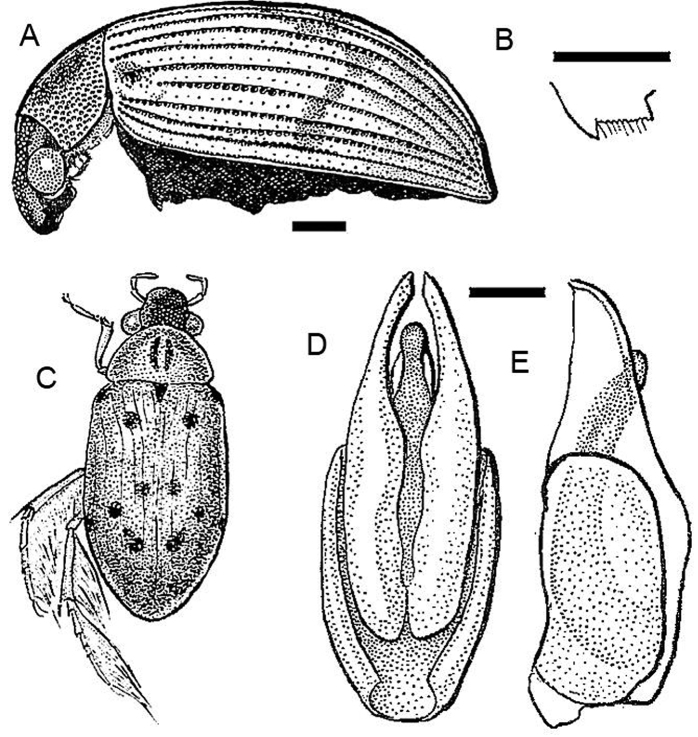
*Berosus auriceps*. **A** lateral habitus (appendages omitted) **B** mesoventral process, lateral **C** dorsal habitus (legs of right side omitted) **D** male genitalia in dorsal aspect **E** idem in lateral aspect. Scale bars= 1 mm. (Reprinted from Oliva 1989).

#### Distribution.

Bolivia, Brazil (Amazonas) and Venezuela (Amazonas).

#### Remarks.

A single series of this species was collected in a rainwater pool formed in exposed rocks in the Orinoco floodplain (Fig. 29). It was taken together with hundreds of specimens of *Berosus garciai* sp. n. *Berosus ruffinus* was previously recorded from Venezuela, also along the Orinoco slightly further downriver (Oliva 1993).

**Figure 26.**
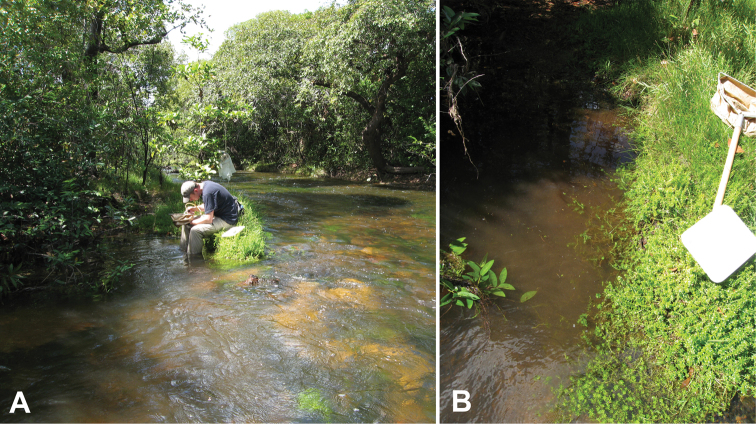
Habitat of *Berosus ambogynus* at the Rio Aguaro near the UCV San Nicolasito Research Station (VZ09-0110-01A) **A** overview of the habitat **B** detail of backwater margin where specimens were collected.

**Figure 27.**
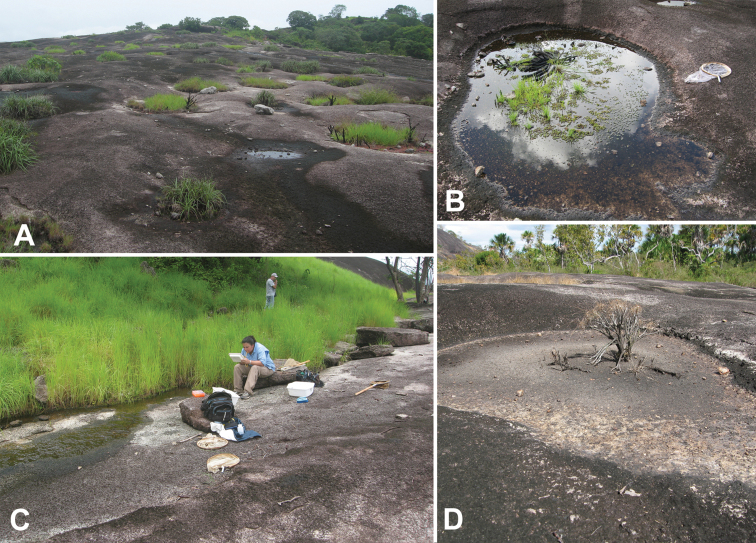
Habitat of Venezuelan *Berosus* spp. at a rock outcrop near Pijiguaos, Bolívar State. **A** Landscape of the rock outcrop in the wet season **B–C** examples of rock pools and seepage on the outcrop in the wet season **D** example of a rock pool on the outcrop in the dry season.

**Figure 28.**
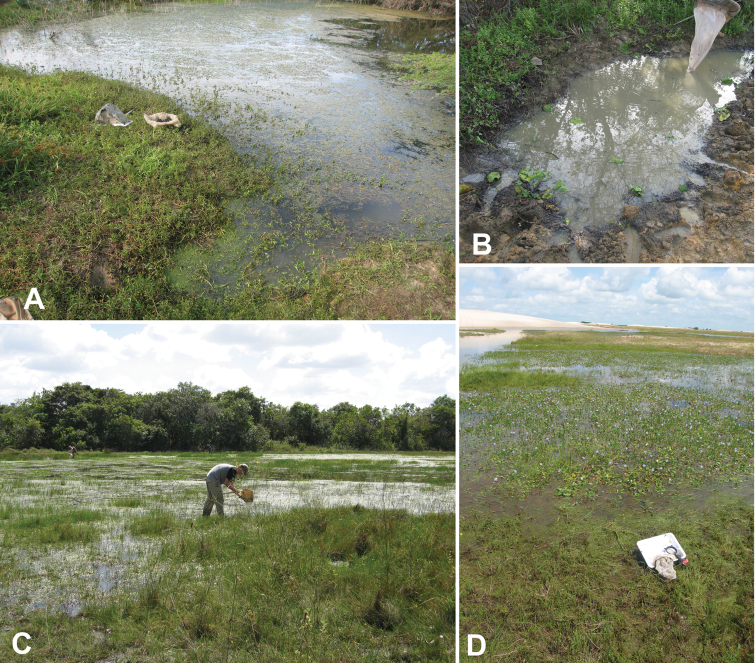
Habitat of Venezuelan *Berosus* spp. **A** Falcón State (VZ10-0710-03B) **B** Falcón State (VZ09-0710-03C) **C** Guárico State (VZ09-0109-01X) **D** Apure State, Medanos de la Soledad (AS-07-004), type locality of *Berosus capanaparo* sp. n..

**Figure 29.**
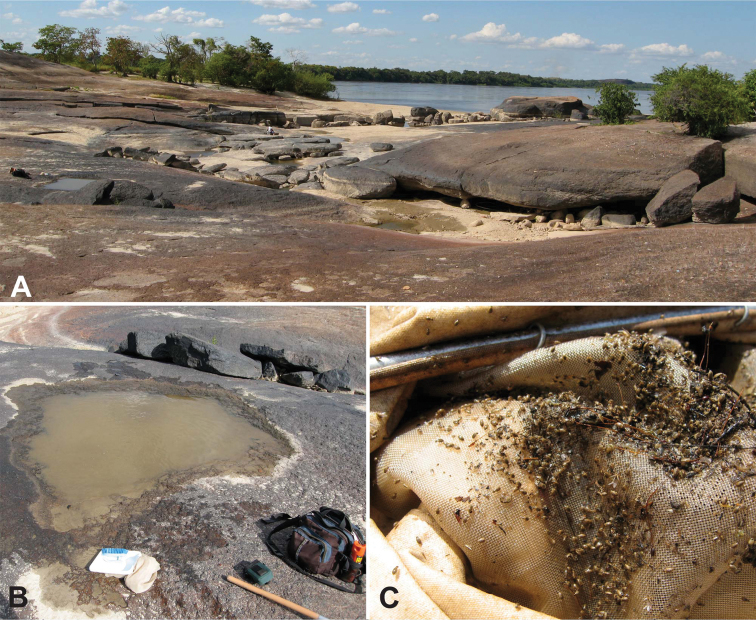
Habitat of *Berosus garciai* and *Berosus ruffinus*; all photos represent collecting event VZ09-0114-02A, Amazonas State, floodplain of the Rio Orinoco **A** general habitat overview **B** rock pool that contained thousands of *Berosus* specimens **C** *Berosus* specimens (nearly all *Berosus garciai*) take flight after being caught in the rock pool.

### Berosus repertus

Oliva & Short sp. n.

urn:lsid:zoobank.org:act:3BB567C6-19EF-4F5B-B014-0930CC57CCE7

http://species-id.net/wiki/Berosus_repertus

Figs 22B , 23B

#### Type material.

**Holotype** (male): “VENEZUELA: Guarico State/ 8°31.705'N, 66°22.602'W, 145 m/ c. 65 km S. Las Mercedes,/ 9.i.2009; leg. Short, Miller/ García & Camacho; large/ lagoon w/veg.; VZ09-0109-01X”, “[barcode]/ SM0844916/ KUNMH-ENT”, “HOLOTYPE/ BEROSUS/ repertus sp. n./ des. Oliva & Short 2010” (MIZA). **Paratypes (13): VENEZUELA: Apure State:** Medanos de la Soledad, 7°20.175'N, 67°43.868'W, 49 m, marshy area, 17.i.2009, leg. Short, Camacho, Miller, VZ09-0117-02X (1 ex., SEMC). **Barinas State:** 10 km NE Barinas, 23.ii.1960, leg. P. & P. Spangler (9 exs., MIZA, SEMC, USNM). **Guárico State:** 20 km S. Calabozo, Rio Orituco, 8-13.ii.1969, leg. P. & P. Spangler (1 ex., USNM); 15 km S. Calabozo, Lago de Los Patos, 9-13.ii.1969, leg. P. & P. Spangler (1 ex., USNM); 32 km SW. Calabozo, 11.ii.1969, leg. P. & P. Spangler (1 ex., USNM).

#### Diagnosis.

Similar to *Berosus palposus* Knisch, 1921 form Brazil (Mato Grosso) in the shape of the male genitalia, but with a longer basal piece. The Brazilian species has remarkably long maxillary palpi, with the apical palpomere elongate, cylindrical and entirely dark, while the palpi of *Berosus repertus* are dark only on the apical one-fourth of the apical palpomere.

#### Description.

Body length: 4.1–4.5 mm. Shape elongately oval in dorsal aspect, moderately convex in lateral aspect. Eyes prominent. Labrum testaceous, melanic at base. Clypeus testaceous on the anterior margin, elsewhere melanic with strong metallic luster. Frons melanic with metallic luster. Pronotum testaceous with melanic discal spot, divided by a testaceous median line. Scutellum dark. Elytra testaceous with small ill-defined melanic spots. Venter of thorax black. Abdomen dark. Maxillary palpi with apical palpomere darkened on apical one-fourth. Femora testaceous, diffusely darkened at base.

Punctures on clypeus about the size of ommatidia, on frons about twice that size, on the base of frons a little larger, contiguous, rather polygonal than round; surface between punctures micropunctate. Pronotal punctures elliptical, moderately coarse, about twice the size of an ommatidion, spaces equivalent to 1–3 diameters, irregular; on the sides punctures round, finer and spaced by 2–3 times their diameter; between punctures there are numerous micropunctations a little smaller than an ommatidion. Scutellum shining, bearing a few punctures. Elytral striae shallow on disc, bearing punctures smaller than those on the pronotum; striae eight, ninth and tenth reduced to rows of punctures on anterior one-third. Interstriae flat, on disc densely micropunctate and bearing punctures about the same size as those on striae; on outer interstriae punctures obsolete. Elytral apices weakly produced in line with the second interstria. Spine-like hairs on all striae (Fig. 22B).

Mesoventral process laminar, with large anterior tooth, very strongly and angularly curved, directed backwards; concave behind ventral margin; posterior angle rounded, strongly raised, but much less so that anterior tooth. Metaventral process narrow, finely carinate in front of median depression; posterolateral angles produced into rounded laminae, posterior angle not raised. First ventrite carinate only between metacoxae, with moderately large, rounded lateral depressions. Fifth ventrite with shallow apical notch, the bottom of which bears two triangular teeth. Lateral margins of abdominal ventrites smooth.

Maxillary palpi elongate, slender; second palpomere longer than fourth, fourth longer than third. Basal pubescence on three-quarters of mesofemora and of metafemora, limit convex. Protarsus of male with first and second tarsomeres strongly thickened, with large adhesive soles, the first about twice as long as the second. Claws weakly arched, angular at base.

Male genitalia (Fig. 23D): basal piece three-fifths of total length, about two and a half times as long as wide. Parameres long, parallel-sided, broadly rounded at apices, with a rather short row of hairs which takes up about half of the unencased part of the parameres. Median lobe a little longer than as parameres, subcylindrical, rounded at apex, with long, fine, weakly raised tergal ridge and a rather small, but distinct sternal opening.

#### Etymology.

From Latin *reperio*, “to find”, alluding to the fact that this species was discovered only after dissecting out the male genitalia.

#### Distribution.

Venezuela (Apure, Barinas, Guárico).

#### Remarks.

Most specimens were taken from standing waters.

### Berosus tramidrum

Oliva & Short sp. n.

urn:lsid:zoobank.org:act:B004430C-D056-4B24-9CBD-18F8B10D7962

http://species-id.net/wiki/Berosus_tramidrum

Figs 14D , 15E

#### Type material.

**Holotype** (male): “VENEZUELA: Guarico State/ 8°59'1.0"N, 65°44'18.8"W, 110 m/ nr. Socorro; 29.vii.2008/ leg. A. Short & M. Garcia/ AS-08-050; Muddy ditch”, “[barcode]/ SM0827667/ KUNHM-ENT”, “HOLOTYPE/ BEROSUS/ tramidrum sp. n./ des. Oliva & Short 2010” (MIZA). **Paratypes (3):** **VENEZUELA: Bolívar State:** ca. 20 km E. Maripa, 7°26'23.2"N, 64°57'5.6"W, 45 m, grassy flooded area, 5.viii.2008, leg. Short & García, AS-08-074 (2 exs., MIZA, SEMC). **Guárico State:** nr. Socorro, 8°59'1.0"N, 65°44'18.8"W, 100 m, muddy ditch, 29.vii.2008, leg. Short & García, AS-08-050 (1 ex., SEMC).

#### Diagnosis.

This species keys to *Berosus marquardti* Knisch, 1921 in the key by Oliva (1993). It differs by the genitalia, especially the median lobe, which in the new species is longer than the parameres and very strongly thickened at the apex (Fig. 15D), and also by the shape of the carina on the first apparent ventrite, which in the new species is much more strongly raised, very strongly convex on anterior two-thirds, behind lower but raised into a separate curve, and by the margins of the fifth ventrite, crenulate in *Berosus marquardti*, coarsely dentate in the new species (Fig. 15D). Otherwise the two species are much alike.

#### Description.

Body length 3.3–3.9 mm. Shape short, broad, convex. Labrum, clypeus and small area of frons close to suture testaceous; most of frons melanic, in typical series reddish. No metallic luster. Pronotum testaceous with narrow median melanic spot divided by a median testaceous line. Scutellum melanic. Elytra testaceous with small spots following the usual pattern. Venter weakly melanic (light reddish in typical series). Femora with pubescent portion darkened, glabrous portion testaceous. Palpi with apical palpomere strongly melanic on distal one-fourth.

Clypeus with dense punctures about the size of one ommatidion, in lateral aspect convex, swollen. Frons even more densely punctured, the punctures twice the size of those on clypeus. Pronotum narrower than humeral humps, bearing contiguous puncture 2–3 times as large as ommatidia, round to polygonal in shape; between these a few deep micropunctures. Elytral striae with deep punctures, on elytral disc about twice as large as those on pronotum, on the sides larger and squarish. Interstriae narrower than striae, convex, the eleventh costate on most of the length, even at the elytral apex. Micropunctation fine but distinct. Humeral angle serrate. Elytral apices narrowly rounded. Spine-like hairs absent.

Mesoventral process short, strongly raised, with hood-like anterior tooth, strongly curved, directed downwards. Posterior angle raised, blunt, more weakly raised than anterior tooth. Metaventral process very wide, posterolateral angles produced into rounded laminae, posterior angle a little less strongly raised, in lateral aspect rounded. First ventrite carinate in its whole length, carina very strongly raised, convex, in anterior two-thirds of sternite further raised, therefore describing a double curve in lateral aspect. Fifth ventrite raised at each side of the deep, narrow apical notch, bidentate at bottom. Ventrites first to fourth crenulate at the sides, fifth coarsely serrate.

Maxillary palpi short, thick. Basal pubescence on two-thirds to three-quarters of mesofemora and two-thirds to three-fifths of metafemora, limit oblique. Protarsus of male almost linear, first and second tarsomeres with small tufts of adhesive hairs; first slightly longer than second. Claws slender, weakly arched, weakly toothed at base.

Male genitalia remarkably small, compressed: basal piece about twice as long as wide. Parameres broadly acuminate, apices strongly curved towards the sternal side. Row of hairs very long, extending along most of the part of the parameron not encased in the basal piece. Median lobe longer than the parameres, strongly curved towards the tergal side, with apex very abruptly and strongly thickened into a rounded club which protrudes entirely from the parameres.

#### Etymology.

The name is an arbitrary association of letters, derived from an anagram of “marquardti”.

#### Distribution.

Venezuela.

#### Remarks.

This species resembles *Berosus marquardti* Knisch, 1921 from which it may be distinguished by the remarkably high, very convex carina on the first apparent ventrite, which in lateral aspect describes a double curve, and by the male genitalia with the median lobe longer than the parameres. In *Berosus marquardti*, the carina is convex only on its middle portion and the median lobe is rather shorter than the parameres, with the apical swelling weaker.

### Berosus truncatipennis

Castelnau, 1840

urn:lsid:zoobank.org:act:C2EBCCA6-8CC0-4195-865B-BE4124552305

http://species-id.net/wiki/Berosus_truncatipennis

Fig. 21A

Berosus truncatipennis Castelnau, 1840: 56.
Berosus truncatipennis Castelnau: Oliva, 1989: 184.

#### Material examined

**(269):** **VENEZUELA:** **Anzoátegui State:** 09°06'42.6"N, 64°09'20.2"W, 228 m, Permanent pond, 12.viii.2009, Cordero, VZ09-812-06A (1 ex.); 09°19'41.4"N, 64°07'07.1"W, 289 m, Temporal rain pond, 13.viii.2009, leg. Cordero, VZ09-813-07A (1 ex.); 09°16'34.6"N, 64°13'39.3"W, 259 m, Temporal pond aside the road, 14.viii.2009, leg. Cordero, VZ09-814-12A (4 exs.); 09°17'16.3"N, 64°13'39.1"W, 274 m, Temporal pond aside the road, 15.viii.2009, leg. Cordero, VZ09-815-12A (2 exs.). **Apure State:** S. San Fernando, 7°52.159'N, 67°29.260'W, 52 m, 3.i.2006, light on top of house, leg. Short, AS-06-006 (1 ex.); ca. 18 km S. San Fernando, 7°33.869'N, 67°38.456'W, 50 m, marsh along road; 4.i.2006, leg. Short, AS-06-008 (6 exs.); Road between Rio Capanaparo and Rio Cinaruco; Medanos de la Soledad, 7°20.175'N, 67°43.868'W, 49 m, marshy area, 17.i.2009, leg. Short, Camacho, Miller, VZ09-0117-02X (2 exs.); Rt. 19, E. Apurito, 7°54.823'N, 68°25.782'W, shaded “pond” by road, 18.i.2009, leg. Short, García, & Camacho, VZ09-0118-01X (4 exs.); between “La Ye” & Bruzual, 7 38.660'N, 69 18.004'W, 90 m, lagoon, 18.i.2009, Short, Camacho & García, VZ09-0118-03X (1 ex.). **Aragua State:** “Venez. Cagua/Edo. Aragua/28-VI-1961 Bordon.” (32 exs., MSUC). **Delta Amacuro State:** 09°08'14.0"N, 61°57'31.3"W, 6 m, Bank of a swamp formed by Orinoco flooding, 26.viii.2009, leg. Cordero, VZ09-826-27A (1 ex.); 09°06'03.7"N, 61°58'44.9"W, 4 m, Bank of a swamp formed by Orinoco flooding, 27.viii.2009, leg. Cordero, VZ09-827-29A (3 exs.); 09°08'39.8"N, 61°55'46.0"W, 3 m, Permanent grass pond, 27.viii.2009, leg. Cordero, VZ09-827-31A (1 ex.); 09°012'00.1"N, 61°53'21.3"W, 3 m, Flooded pond, 28.viii.2009, leg. Cordero, VZ09-828-35A (2 exs.). **Falcón State:** E. Dabajuro, River at Bridge crossing on Rt. 3, 11°02.305'N, 70°38.467'W, 98 m, Gravel and sand margins of river and associated side pools, 8.vii.2009, leg. Short, Gustafson, & Inciarte, VZ09-0708-01A (1 ex.); Médanos de Coro, pond in dunes, 11°26.215'N, 69°40.112'W, 8 m, pond in dunes, 9.vii.2009, leg. Shepard, VZ09-0709-03Z (13 exs.); SE of Tocopero, 11°26.922'N, 69°13.109'W, 12 m, Puddle in roadside ditch and culvert, 10.vii.2009, leg. Sites, VZ09-0710-03S (7 exs.); same locality but leg. Short et al., VZ09-0710-03B (23 exs.); same locality but leg. Short et al., VZ09-0710-03C (53 exs.). **Guárico State:** Corozo Pando (8 km N.), 17–18.vi.1984, leg. F.W. Eiland & V. Linares, blacklight (11 exs.); 15 km S. Calabozo, 9–13.ii.1969, Lago de los Patos, leg. P. & P. Spangler (11 exs.); 20 km S. Calabozo, 8–13.ii.1969, Rio Orituco, leg. P. & P. Spangler (2 exs., USNM); 44 km S. Calabozo, Hato Masaguaral, 6.iii.1986, P. Spangler & S. Beaujon, Collection # 31 (9 exs., USNM); ca. 35 km N. San Fernando, 8°11.578'N, 67°36.287'W, 53 m, marshy area by road, 10.ix.2007, leg. A. Short, AS-07-002 (1 ex.); nr. Socorro, 8°59'1.0"N, 65°44'18.8"W, 100 m, muddy ditch, 29.vii.2008, leg. Short & García, AS-08-050 (1 ex.); N. of Palenque, 9°6.794'N, 66°59.595'W, 152 m, stream at road crossing, 8.i.2009, leg. Short, García, Miller, Camacho, Joly, VZ09-0108-03X (3 exs.); on side road ca. 10 km E. Mantecal, 7°37.298'N, 69°3.679'W, 83 m, marshy area and pool by road, 18.i.2009, leg. Short, García, & Camacho, VZ09-0118-02X (4 exs.); ca. 65 km S. Las Mercedes, 8 31.705'N, 66 22.602'W, 145 m, large lagoon with vegetation, 9.i.2009, leg. Short, Miller, García, & Camacho, VZ09-0109-01X (2 ex.); W. of Las Mercedes, 9°5.067'N, 66°28.500'W, 153 m, 8.i.2009, leg. Short & Miller, roadside pond, VZ09-0108-04A (1 ex.); San Nicolasito Field Station, 8°8.677'N, 66°24.263'W, 50 m, pool without fish, 10.i.2009, leg. Miller & García, VZ09-0110-03A (2 ex.). **Portuguesa State**: Guanare, 26.ii.1969, leg. P. & P. Spangler (1 ex., USNM); Rio Guanare, N. of Guanare, 8°25.773'N, 69°35.202'W, 185 m, side rocky pools, 19.i.2009, leg. Short, VZ09-0119-02C (7 exs.). **Sucre State:** Finca Vuelta Larga, 10°30.075'N, 63°06.217'W, 10 m, muddy puddle in road with detritus, 29.i.2010, leg. Short, García, Joly, VZ10-0129-03A (2 exs.). **Zulia State**: 40 km SW Machiques, Rio Tucuco, 3.vii.1984, blacklight trap, leg. Eiland & Linares (1 ex., USNM); Farm of the Universidad del Zulia (Ana Maria Campo), W. of Maracaibo, stock tank, 20.i.2006, leg. Short, AS-06-037 (1 ex.). When not indicated, material is distributed between MIZA, MALUZ, SEMC, and NMW.

#### Distribution.

Widespread in tropical and subtropical South America, including NE Argentina, Bolivia, Brazil, Paraguay, and Venezuela (Anzoátegui, Apure, Aragua, Delta Amacuro, Falcón, Guárico, Portuguesa, Sucre, Zulia). Records for Central America and the Caribbean need confirmation. Previously recorded from Venezuela (Apure) by Oliva (2002).

#### Remarks.

The apex of the median lobe is a fine membranous dome, which may be considerably deformed in dry-mounted specimens. See discussion of *Berosus megaphallus* sp. n.

### Berosus wintersteineri

Knisch, 1921

http://species-id.net/wiki/Berosus_wintersteineri

Berosus (Berosus) wintersteineri Knisch, 1921: 14.
Berosus wintersteineri Knisch: Oliva (1989: 91).

#### Material examined

**(10): VENEZUELA:** **Guárico State:** Hacienda Nicolasito, 61 m, 8°8'20"N, 66°24'32"W, 15–17.vi.2000, leg. M. Gaiani, P. Freitag, & Q. Arias (10 exs., MIZA, SEMC).

#### Distribution.

Brazil and Venezuela (Guárico).

### Berosus zimmermanni

Knisch, 1921

http://species-id.net/wiki/Berosus_zimmermanni

Berosus (Berosus) zimmermanni Knisch, 1921: 15.
Berosus zimmermanni Knisch: Oliva (1989: 89).

#### Material examined

**(8). VENEZUELA: Apure State:** Hato El Frio, Fundo Ceibote, 20.v.1975, leg. C. Rosales (4 exs., MIZA, SEMC); Fundo La Florida, Rio Quitaparo, 7°05'N, 68°26'W, 42 m, 11–14.vi.1999, leg. E. Osuna & A. Chacon (1 ex., MIZA); same data but 16–18.v.1999 (1 ex., SEMC). **Portuguesa State:** San Nicolas Experimental Station, 56 km from Guanare, 180 m, 11.v.1975, light trap (1 ex., MIZA). **Tachira State:** San Joaquin de Navay, 400 m, 21–22.vi.1979, leg. A. Chacon (2 exs., MIZA). Representative specimen will be deposited in NHW, NMPC, and USNM.

#### Distribution.

Argentina, Brazil, Paraguay, and Venezuela (Apure, Portuguesa, Táchira). New record for Venezuela

#### Remarks.

Although species is known from several localities in the southwestern Llanos and in the foothills of the Andes, no new specimens have yet been collected during recent fieldwork. This species is remarkable by the claws, which are bidentate in all the legs and in both the sexes.

Incertae sedis

### Berosus sp. A

#### Material examined

**(1):** **VENEZUELA:** **Bolívar State:** Los Pijiguaos, 6°35.617'N, 66°49.238'W, 80 m, morichal/rock outcrop, 16.ix.2007, leg. Short, García, & Joly, AS-07-015 (1 ex., MIZA).

#### Distribution.

Venezuela (Bolívar).

#### Remarks.

A single female, small in size, with the pronotum entirely melanic with metallic sheen, densely and deeply punctate.

### Key to species of Venezuelan *Berosus*

Some species from neighbor countries have been added for comparison.

**Table:** 

1	Dorsal sculpture very coarse (Figs 10, 14). Punctures on pronotum 4–6 times the size of ommatidia; punctures on elytral striae larger than those on pronotum. Interstriae on elytral disc never wider than striae. Lateral margins of ventrites crenulate in most species (Figs 10B, 15B–D); first ventrite carinate on its entire length or a large part of it. Protarsus of male with basal tarsomeres not thickened, bearing a few modified hairs, but not the distinct adhesive soles found in most species of the genus (See Oliva 1989, Fig 41. for the protarsus of *Berosus holdhausi* Knisch)	2
–	If sculpture is coarse, the elytral interstriae are distinctly wider than the striae, at least on elytral disc, and the punctures on the striae are not larger than those on the pronotum (Figs 5, 16, 22). Protarsus of males always with a distinct sole of adhesive hairs at least on the basal tarsomere, usually on the two basal ones; the two basal tarsomeres thickened (Oliva 1989, Figs 127, 213, 332)	9
2(1)	Inner elytral striae (on disc) narrower than interstriae (Fig. 10)	3
–	Inner elytral striae as wide as interstriae or wider	4
3(2)	Interstriae strongly convex, except the inner one (first interstria) on the anterior half; outer (eleventh) interstria costate, overhanging elytral margin on anterior half (Fig. 10A). Mesoventral process with anterior tooth strongly curved, slightly swollen, directed downwards; posterior angle raised, much lower than anterior tooth. Male genitalia with basal piece about half of total length. Parameres gradually acuminate; median lobe with spindle-shaped apical swelling (Fig. 10C)	*Berosus consobrinus* Knisch, 1921
–	Elytral interstriae flat, more deeply and coarsely punctate. Mesoventral process with anterior tooth weakly curved, laminar, directed downwards and backwards; posterior angle raised into a blunt tooth nearly as long as the anterior one. Male genitalia with basal piece shorter than half of total length. Median lobe of male genitalia with abrupt subapical swelling, and a short slender apex	*Berosus wintersteineri* Knisch, 1921
4(2)	Mesoventrite entirely laminar	5
–	Mesoventrite with anterior tooth hooded	6
5(4)	Dorsum blackish with weak metallic sheen on head including labrum and pronotum, the latter testaceous on lateral margins and anterior angles. Elytra shading into dark brown at the apices and outer margins (Fig. 14B). Mesoventral process with anterior tooth weakly curved, directed posteroventrally; posterior angle weakly raised. Apical palpomere of maxillary palpi dark on apical one-third. Femora with pubescent portion darkened, glabrous portion testaceous. Male genitalia as in Fig. 15A	*Berosus ebeninus* sp. n.
–	Dorsum testaceous, yellowish in dead specimens (may be greenish when specimens are alive), with elytra slightly lighter than head and pronotum: elytral spots reddish (Fig. 14A). Mesoventral process with anterior tooth straight, directed posteriorly; posterior angle raised, nearly as much as anterior tooth. Apical palpomere dark only at apex. Femora with pubescent portion not darker than glabrous portion. Male genitalia as in Fig. 15C	*Berosus corozo* sp. n.
6(4)	All the claws bifid (Oliva 1989: Fig. 55). Dorsum blackish, without any metallic luster or sheen. Body shape elongate. Median lobe of male genitalia with weak subapical swelling and a long apex	*Berosus zimmermanni* Knisch, 1921
–	Claws dentate at base. Dorsum testaceous, usually with small darker areas. Body shape short and wide (Fig. 14C, D)	7
7(6)	Dorsum of head melanic with strong metallic luster (Fig. 14C). Tibiae dark at apex. Tarsi often dark (not noticeably so in Venezuelan specimens). Mesoventral process with large, strongly curved anterior tooth and small acute posterior tooth (Oliva 1989: Fig. 39). Male genitalia: basal piece a little longer than half of total length, parameres gradually acuminate; median lobe a little shorter than parameres, otherwise similar to *Berosus ebeninus* sp. n. but more weakly curved	*Berosus holdhausi* Knisch, 1921
–	Head without metallic luster. Mesoventral process with straight, rather slender anterior tooth, posterior angle not raised. Tibiae not dark at apex in Venezuelan specimens	8
8(7)	Carina of the first ventrite moderately raised, convex only in anterior half (Oliva 1989: Fig. 50). Parameres with short subapical row of hairs. Median lobe moderately swollen at apex, a little shorter than the parameres, weakly curved towards sternal aspect, moderately swollen at apex, the swelling encased in parameres (Oliva 1989: Fig. 52)	*Berosus marquardti* Knisch, 1921
–	Carina of the first ventrite very strongly raised, the anterior half more so, in lateral aspect describing two convex curves. Median lobe rather longer than parameres, very strongly swollen at apex, blunt. Parameres with long row of hairs that extends along most of paramere (Fig. 15D). Median lobe distinctly longer than the parameres, strongly curved, strongly swollen at apex, the swelling free of the parameres	*Berosus tramidrum* sp. n.
9(1)	First ventrite carinate behind the metacoxae, without lateral depressions. Small species (length usually less than 4 mm), without any metallic luster on dorsum, without spine-like hairs on elytra (Figs 6A–B, 18, 19). Elytral apices never bispinous as in Fig. 2. Protarsus of males with adhesive soles on two basal tarsomeres, usually not longer than in females. Maxillary palpi usually short and thick in both sexes (*sticticus*-complex)	10
–	First ventrite carinate only between metacoxae; if carinate behind them, the head has a metallic luster (Fig. 5) or the shape is elongate or the elytral apices are produced as in Figs 1, 24 or bispinous as in Figs 2, 22A	17
10(9)	Metaventral process with posterior angle raised into a small lamina which is convex in lateral aspect (Oliva 1989: Fig. 85)	11
–	Metaventral process with posterior angle not raised into a lamina, although it may be carinate (compare Oliva 1989: Fig. 183)	12
11(10)	Outer elytral striae (ninth, tenth and eleventh) flat (Fig. 19A). Fifth ventrite of males with two acute raised teeth, one behind the other, in front of the apical notch (Fig. 20A). Mesoventral process with anterior tooth nearly straight, directed downwards. Fifth ventrite of males with a small raised tooth in front of the apical notch (Fig. 20A). Protarsus of male very short and thick; first tarsomere hardly longer than second, both weakly swollen; fourth tarsomere nearly straight. Male genitalia with basal piece about two-thirds of total length. Parameres curved, with subapical emargination, apices narrow; subapical row of hairs short, but the hairs themselves long (Fig. 20A)	*Berosus llanensis* sp. n.
–	Outer interstriae strongly convex (but not costate), especially on the anterior half of elytron behind the humeral hump (Fig. 13). Mesoventral process with anterior tooth curved, directed downwards and backwards. Fifth ventrite of males without raised teeth in front of the apical notch (Fig. 13). Protarsus of male longer than that of female; first tarsomere longer than second; fourth tarsomere curved. Basal piece long, about four-fifths of total length (Fig. 13). Parameres strongly curved, without subapical emargination, but narrowing to the acuminate apices	*Berosus guyanensis* Queney, 2006
12(10)	Labrum deep black even in light-colored specimens. Elytral disc bearing two pairs of sutural spots, but no spots on the fourth interstria (Fig. 6B). Outer interstriae weakly convex	13
–	Labrum usually testaceous; black only in very dark specimens	14
13(12)	Interstria tenth wider than eleventh (Fig. 6B). Mesoventral process with anterior tooth directed downwards and backwards. Metaventral process with posterior angle carinate, not more strongly raised than the posterolateral angles, which are produced into laminae. First ventrite carinate on anterior two-thirds. Apical notch of fifth ventrite with bottom produced into a pair of teeth (Fig. 7B). Median lobe of male genitalia as long as parameres (Fig. 7B)	*Berosus garciai* sp. n.
–	Interstria tenth not wider than eleventh (Fig. 18). Mesoventral process with anterior tooth directed downwards. Metaventral process with posterolateral angles hardly produced, posterior angle not carinate. First ventrite carinate on its whole length. Apical notch of fifth ventrite with bottom produced into a single small tooth (Fig. 18). Parameres with short subapical row of hairs (Fig. 18). Median lobe a little longer than parameres	*Berosus jolyi* sp. n.
14(12)	Head with a dark triangle taking up most of the frons and the middle of the clypeus, often black with slight metallic sheen (Fig. 6A). Pronotum with extensive reddish or dark areas, only the anterior angles testaceous. Outer interstriae flat (Fig. 6A). First ventrite carinate on its whole length. Male genitalia weakly compressed, asymmetrical (Fig. 7A)	*Berosus asymmetricus* sp. n.
–	Clypeus entirely testaceous; frons usually dark only at base; dorsum testaceous (elytra may be greenish) with small darker areas (Figs 18, 19). At least interstriae ninth and tenth convex. Male genitalia symmetrical, strongly compressed	15
15(14)	Outer elytral interstriae costate (Fig. 19B). Male protarsus with very strongly thickened basal tarsomere. Male mesotarsus with inner claw shorter and more strongly arched than outer claw. Male genitalia long, basal piece about half of total length. Parameres with the free sternal margin long and nearly straight (Fig. 20B)	*Berosus olivae* Queney, 2006
–	Outer elytral interstriae not costate (as in Fig. 19A). Male protarsus with first tarsomere not thicker than second. Parameres with the free sternal margin short, convex	16
16(15)	Antennal cupula normal (hardly wider than pubescent antennomeres forming the club). Eleventh interstria convex, about as strongly raised as interstria ten. Mesoventral process with anterior tooth weakly curved, slender, acute, directed downwards and backwards. Apical notch of fifth ventrite with a bifid tooth, in males a small carina in front of this. Basal piece about three times as long as wide, three-quarters of total length. Parameres strongly curved towards the sternal side, apices abruptly narrowed, directed sternally; row of hairs moderately long	*Berosus festivus* Berg, 1885
–	Antennal cupula enlarged (about twice as long as the width of club). Tenth interstria convex, more strongly raised than flat eleventh interstria on the posterior half of the elytra. Mesoventral process with anterior tooth strongly curved. Apical notch with bottom very weakly produced into a triangle. Basal piece two-thirds of total length. Parameres rounded in apical half, apices not narrowed, but with a minute emargination that determines two teeth	*Berosus duquefi* Queney, 2006 (French Guiana)
17(9)	Basal pubescence of meso- and metafemora oblique as in Fig. 14C. Elytra without spine-like hairs (as in e.g. Fig. 22). First ventrite carinate between metacoxae, without lateral depressions. Head entirely melanic on dorsum, with metallic luster. Dorsum of head, pronotum and elytral striae coarsely punctate. Basal piece of male genitalia encasing only the base of the acuminate parameres	*Berosus nervulus*-complex (no species found in Venezuela to date)
–	Basal pubescence of meso- and metafemora with limit transverse or briefly oblique. First ventrite carinate only between metacoxae; if carina longer, then dorsum of head without metallic luster (Fig. 11), or elytra bearing spine-like hairs, or both.	18
18(17)	Protarsi of males with modified hairs ventrally forming an adhesive sole on the basal tarsomere only (Oliva 1989: Fig. 127). Metallic luster on dorsum of head. Body shape broad but not very convex in lateral aspect (Figs 16A–B, 25C). Spine-like hairs only on the posterior third of the elytral margins, but may be difficult to see. Metaventral process large, strongly raised (Fig. 25A–B). Abdominal ventrites with lateral margins serrate, the first ventrite carinate on anterior half, with small glabrous areas or rudimentary lateral depressions; fifth ventrite raised at the sides of the apical notch, which bears a large bifid tooth at the bottom. Male genitalia cylindrical (Figs 17A–B, 25D, E); parameres parallel, acuminate or bluntly narrowed; median lobe thick, curved towards the tergal side, or very thick and straight (*Berosus auriceps*-complex)	19
–	Protarsi of males with adhesive soles on the two basal tarsomeres. If the bottom of the apical notch bears two teeth, then the lateral margin of the ventrite is smooth	21
19(18)	Mesoventral process with anterior tooth slightly hooded, large, the posterior part in lateral aspect rectangular (Fig. 25B). Pronotum with discal black spot divided by a testaceous median line and usually without any metallic luster. Interstriae wide, flat, the outer ones slightly convex, bearing punctures a little smaller than the strial ones, much finer on the posterior third of the elytron. Humeral humps with small rounded dark spots on sixth interstria, interstriae 7–9 testaceous (Fig. 25A, C). Size large (length 5.0–6.7 mm). Body shape elongate. Parameres acuminate, apices curved inwards. Median lobe much shorter than parameres, slender, rounded at apex, curved towards the tergal side (Fig. 25D, E) (See also Oliva 1989: Figs 124–130)	*Berosus auriceps* Boheman, 1859 (Argentina, Paraguay, Brazil)
–	Mesoventral process entirely laminar. Humeral humps with small spots on sixth interstria, also dark spots just below humps taking up at least interstriae 9 (Fig. 16A, B). Size moderate (3.4–4.6 mm). Body shape short, broad	20
20(19)	Labrum black. Pronotum with extended black spot, with metallic sheen. Elytra with small black spots, including a supplementary pair below each humeral hump, on interstria nine (Fig. 16B). Punctures on elytral striae overflowing outwards. Apical palpomere of maxillary palpi with apical darkening extensive, covering distal third of palpomere (Fig. 16B). Fifth ventrite with shallow notch, bottom of this bearing a rounded bifid tooth. Male protarsus weakly thickened at base, with basal tarsomere about twice as long as second. Basal piece a little less than a half of total length. Parameres with subapical row of hairs short. In tergal aspect parameres gently curved inwards. Median lobe as long as parameres, roughly pear-shaped, with a constriction (Fig. 17B)	*Berosus ornaticollis* sp. n.
–	Labrum melanic at base, testaceous on anterior portion. Pronotum with a discal melanic spot with metallic sheen, about two-fifths of total pronotal width. The spots below humeral humps take up interstriae eight, ninth and part of the tenth (Fig. 16A). Punctures on elytral striae not overflowing outwards (except for a short stretch of striae 6–8 on the posterior half of elytra). Male protarsus with basal tarsomere three times as long as the second. Maxillary palpi with apical palpomere melanic on distal quarter (Fig. 16A). Bottom of apical notch produced into two straight sharp teeth. Basal piece a little more than half of total length. Parameres with subapical row of hairs rather long. In tergal aspect, parameres elbowed rather than curved inwards. Median lobe a little shorter than parameres, thick (Fig. 17A)	*Berosus humeralis* sp. n.
21(18)	Elytra without spine-like hairs. Mesoventral process short, wide, with posterolateral angles always produced. First ventrite without lateral depressions. Dorsal sculpture moderate to coarse; outer elytral striae more or less deepened over the large lateral dark spot, sometimes widened and deflected at this point (Oliva, 1989, Fig. 172). Basal piece of male genitalia encasing the greater part of the distal ones. Parameres forming a dihedral angle	*Berosus adustus*-complex. No species known from Venezuela. One widespread South American species, *Berosus asphaltinus* Knisch, 1922, might extend to Venezuela
–	Head without metallic luster; if head with metallic luster, then elytra with spine-like hairs (Fig. 22), or elytral spots forming a band on anterior half of elytron as in Fig. 5	22
22(21)	Body shape elongate, weakly convex (Fig. 22A–B). Head with metallic luster, but labrum (except for distal margin) and anterior margin of clypeus testaceous. All the elytral interstriae bear spine-like hairs (Fig. 22A–B). Pronotal punctures coarse, dense; pronotal ground micropunctate. First ventrite carinate only between the posterior coxae, in most species with small lateral depressions. Maxillary palpi very long and slender, especially in males. Size moderate to small (length 3.0–4.7 mm). Male genitalia cylindrical. Parameres acuminate, parallel. Median lobe thick, weakly sinuate or straight, with a tergal ridge and a large sternal opening (Fig. 23)	*Berosus patruelis*-complex 23
–	Body shape broad (Fig. 5), if shape elongate then pronotal sculpture not coarse (see Fig. 2A–D). Sternal margins of parameres always forming a dihedral angle, although this may not be clear when the genitalia are strongly compressed (Figs 4, 8)	24
23(22)	Elytral apices not emarginate, weakly produced. Pronotum with rather fine, elliptical punctures. Fifth ventrite with shallow apical notch, the bottom of which bears two triangular teeth (Fig. 23B). Protarsus of male strongly dilated at base. Male genitalia with basal piece about three-fifths of total length. Median lobe not dilated subapically (Fig. 23B)	*Berosus repertus* sp. n.
–	Elytral apices emarginate, in some individuals narrowed and truncated (Fig. 22). Pronotal punctures dense (spaces smaller than diameters). Fifth ventrite with very wide, very shallow apical emargination (Fig. 23A). Male genitalia with short basal piece; median lobe moderately shorter than parameres, weakly dilated at apex, with short tergal ridge (Fig. 23A)	*Berosus patruelis* Berg, 1885
24(22)	Elytral striae ninth and tenth often reduced to rows of punctures at base. Spine-like hairs at least on interstria eleventh. Elytral apices produced, weakly emarginate or both, not bispinous. Pronotum with round, moderately coarse punctures and coarse, dense micropunctation. Male genitalia compressed. Parameres long, dilated at apex; median lobe cylindrical	*Berosus chalcocephalus*-complex 25
–	Elytral apices either rounded or bispinous, if produced, then there is no micropunctation on the pronotum, or the first ventrite is carinate behind the metacoxae, with large lateral depressions	27
25(24)	Elytral apices produced into large triangles, acute. Elytral interstriae weakly convex. Mesoventral process with small anterior tooth. Metaventral process with posterior and posterolateral angles produced into laminae. Apical notch of ventrite fifth with bottom bidentate. Dorsum reticulate in females. Length 4.7 mm (female holotype)	*Berosus navatus* d’Orchymont, 1940 (Brazil: Pernambuco)
–	Elytral apices blunt, sometimes weakly emarginate. Elytral interstriae flattened. Metaventral process with posterolateral angles not or weakly produced, posterior angle not raised into a lamina (Oliva 1989: Fig. 209). Parameres with inner membranous appendices (Oliva 1989: Figs 215, 225)	26
26(25)	Length 2.9–3.8 mm. Outer elytral striae deeply impressed. Fifth ventrite with bottom of the notch bidentate, in males hidden by a carinate tooth (see Oliva 1989: Fig. 235); another such tooth on the fourth ventrite. Parameres subapically dilated, abruptly acuminate at apices. Appendices large, nearly as long as the parameres. Median lobe as long as the appendices	*Berosus inpa* Oliva, 1993 (Brazil: Amazonas)
–	Length usually over 4.0 mm. The three outer elytral striae reduced to rows of punctures on base of elytra. Fifth ventrite with bottom of apical notch produced into a pair of rounded projections; no carinate teeth on ventrites. Parameres dilated apically into a trapeze. Membranous appendices much shorter than the parameres. Median lobe a little longer than appendices (Oliva 1989: Figs 214, 125)	*Berosus pallipes* Brullé, 1841
27(24)	First ventrite without distinct lateral depressions. Spine-like hairs on all elytral interstriae (South American species) or only on the eleventh. Body shape weakly convex, rather narrow, but not elongate (Fig. 12). Male genitalia cylindrical or compressed. Basal piece long, encasing at least the basal half of the acuminate parameres. Median lobe subcylindrical, weakly sinuate to straight (Oliva 1989: Figs 229, 232, 240, 237–240)	*Berosus corumbanus*-complex 28
–	First ventrite with distinct lateral depressions	30
28(27)	Apical emagination of fifth ventrite bearing a pair of straight, not divergent teeth (Fig. 9). Lateral margins of ventrites smooth. Dorsum of head testaceous, with dark median triangle. Basal piece three-quarters of total length. Parameres strongly acuminate in distal half, with row of hairs along one-third of their length (Fig. 9)	*Berosus castaneus* sp. n.
–	Apical emargination of fifth ventrite bearing at the bottom a pair of contiguous, divergent teeth; males with a carinate tooth in front of notch (Fig. 12). Lateral margins of ventrites serrated	29
29(28)	Dorsum of the head melanic with metallic luster. Basal tarsomere of male protarsi weakly dilated. Basal piece about half of total length	*Berosus pluripunctatus* Mouchamps, 1963
–	Dorsum of head partly testaceous, without metallic luster (Fig. 12). Hind femora of males angular. Male genitalia with basal piece very long. Parameres abruptly acuminate, row of hairs short, subapical (Fig. 12)	*Berosus geayi* d’Orchymont, 1937
30(27)	Metaventral process with posterolateral angles not produced (*Berosus alternans*-complex), posterior angle not carinate (Fig. 5A, B). Inner elytral interstriae with punctures as coarse as those on striae. Dorsum of head, including labrum, melanic with metallic luster. Elytra with small black spots; additional spots extending between the humeral spot and the first sutural spot, in some specimens forming an oblique dark band (Fig. 5A, B). Length 3.1–4.5 mm. Body shape depressed, not elongate. Maxillary palpi with distal palpomere melanic on distal half. Male genitalia as in Fig. 5D	*Berosus aragua* sp. n.
–	Metaventral process with posterolateral angles more or less produced. Sutural and humeral dark spots usually not connected by distinct additional spots (Fig. 11). If the dorsum of head has a metallic luster, the elytral apices are emarginate (Fig. 2A)	31
31(30)	Body shape elongate as in Fig. 11. Dorsal sculpture moderately dense to sparse; ground micropunctate. Mesoventral process broadened at the level of the posterolateral angles, which are produced. Male genitalia weakly compressed. Parameres acuminate. Median lobe cylindrical, with apex spindle-shaped, weakly swollen (*Berosus obscurifrons*-complex). Dorsum of head without metallic luster, partially testaceous. Spine-like hairs on interstria eleventh only. Elytral apices weakly produced, rounded. Basal piece about one-third of total length of male genitalia. Parameres with long rows of hairs (Oliva 1989: Fig. 272)	*Berosus elegans* Knisch, 1921
–	Body shape not elongate (Fig. 2A–D), or else narrow as in Fig. 1	32
32(31)	Lateral depressions on first ventrite small but usually deep. Dorsum micropunctate, reticulate or alutaceous. Head with or without metallic luster. Body shape depressed, sturdy to slender, with prominent humeral humps (Fig. 2A–D). Male genitalia strongly compressed. Parameres long, apices narrowly rounded (Fig. 4). Median lobe long, cylindrical, straight or weakly curved, apex blunt or rounded	*Berosus reticulatus*-complex 33
–	Lateral depressions on first ventrite large and rounded (see Oliva 1989: Fig. 324). No metallic luster on dorsum. Body shape slender or elongate; humeral humps weakly prominent	38
33(32)	The whole dorsum, including head, reticulate in both sexes, with soft velvet-like sheen, finely punctured. Head melanic with metallic luster (Fig. 2A). Pronotum with large black discal spot. Elytra with small but distinct black spots. Femora testaceous. Fifth ventrite with serrate margin, notch bidentate (Fig. 4C). Basal piece of male genitalia a little less than half of total length (Fig. 4C)	*Berosus reticulatus* Knisch, 1921
–	Head never reticulate. Femora testaceous only on glabrous portion, the pubescent area melanic (Fig. 2D)	34
34(33)	Head entirely melanic, with metallic luster. Margins of fifth ventrite serrate. Sutural angle produced, acute. Basal piece a little less than half of total length. Parameres not emarginate	*Berosus ghanicus* d’Orchymont, 1941
–	At least the clypeus testaceous.	35
35(34)	Frons entirely melanic, sometimes with strong metallic luster (Argentinean specimens), in specimens from Venezuela and Guyana, with weak metallic sheen or even without it. Dorsum of head and pronotum alutaceous, not shining. Body size large (length 5.4–6.3 mm). Fifth ventrite with margins finely serrate, bottom of apical notch bidentate (Fig. 4B). Basal piece about half of total length of male genitalia (Fig. 4B)	*Berosus erraticus* Mouchamps, 1963
–	Usually only the base of the frons melanic. Dorsum of head and pronotum between the punctures usually shining. Fifth ventrite with smooth margins	36
36(35)	Bottom of apical notch of fifth ventrite produced into an arc (Fig. 3). Sutural angle of elytra produced into an acute triangle about two-thirds of the length of the parasutural spine (Fig. 2B). Pronotum without micropunctation between punctures (Fig. 2B). Basal piece about two-fifths of total length	*Berosus ambogynus* Mouchamps, 1963
–	Bottom of apical notch of fifth ventrite with blunt teeth (Fig. 4A). Sutural angle of elytral apex not produced. Pronotum densely micropunctate	37
37(36)	Bottom of apical notch with a small, blunt bifid tooth (Fig. 4A). Ground of pronotum between punctures, shining in both sexes (Fig. 2C). Basal piece very short, less than one-quarter of total length of male genitalia. Median lobe with sub-basal swelling (Fig. 4A)	*Berosus brevibasis* Oliva, 1989
–	Bottom of apical notch with two contiguous rounded teeth. Basal piece one-third of total length. Parameres with a long row of hairs. Median lobe shorter than parameres, weakly curved, without sub-basal swelling (Fig. 8)	*Berosus capanaparo* sp. n.
38(32)	Body shape narrow as in Figs 1 and 24. Male genitalia moderately compressed, simple, parameres without membranous appendices. Median lobe subcylindrical; apex rounded, often swollen (Oliva 1989: Figs 292, 293; see also Figs 300, 305, 311). Males without modifications on legs, except the protarsus, which has the two basal tarsomeres thickened and bearing large soles of adhesive hairs (Oliva 1989: Fig. 310). Spine-like hairs on all the elytral interstriae	*Berosus subtilis*-complex 39
–	Body shape elongate, not narrow. Pronotum always micropunctate between punctures. Male genitalia not compressed, complex, parameres with inner membranous appendices (Oliva 1989: Figs 333, 334; see also Figs 317–320, 325, 236, 342–344, 352, 353). Median lobe simple or complex. Males often with modifications of the legs (Oliva 1989: Fig. 338, 348, 349) or of the claws (Oliva 1989: Figs 339, 340, 347) besides the usual modifications of the protarsus. Venezuelan species with sexual dimorphism in fifth ventrite (Fig. 21A, B)	*Berosus truncatipennis*-complex 40
39(38)	Fifth ventrite without a notch. Elytral apices with semicircular emargination in males, produced and converging in females (Fig. 1). Pronotum finely punctate, ground between punctures micropunctate, smooth in males, reticulate in females. Elytra reticulate in both sexes, shining in males, in females a little matt. Mesoventral process semicircular, weakly serrate, without teeth. Parameres encased in basal piece in basal half, abruptly acuminate in distal ½ of unencased portion, with a short row of long hairs placed about the middle of the length of this portion. Median lobe as long as parameres, slender, sinuate, with abrupt apical dilation, disc-shaped in tergal aspect	*Berosus apure* Oliva, 2002
–	Fifth ventrite with apical notch. Elytral apices produced, rounded in males, acute and convergent in females (Fig. 24). Pronotum and elytra with moderately coarse punctures; no micropunctation. Elytra never reticulate in males. Mesoventral process with large, curved anterior tooth. Male genitalia with parameres encased in basal piece in most of their length, with large, semicircular apical emargination, the row of hairs replaced by a single thick arista. Median lobe much longer than parameres, slender, straight, greatly swollen at apex	*Berosus ruffinus* d’Orchymont, 1946
40(38)	Pronotal micropunctures about one quarter of punctures. Basal piece of male genitalia short, about one-third of total length. Parameres broad, blunt, with a long row of hairs; ventral membranous appendices shorter than the median lobe (Fig. 21A)	*Berosus truncatipennis* Castelnau, 1840
–	Pronotal micropunctures about one sixth of punctures. Basal piece of male genitalia about two fifths of total length. Parameres with a tuft of hairs instead of a row, with membranous appendices a little longer than the median lobe (Fig. 21B). Other external characters as in precedent	*Berosus megaphallus* sp. n.

## Supplementary Material

XML Treatment for Berosus ambogynus

XML Treatment for Berosus apure

XML Treatment for Berosus aragua

XML Treatment for Berosus asymmetricus

XML Treatment for Berosus brevibasis

XML Treatment for Berosus capanaparo

XML Treatment for Berosus castaneus

XML Treatment for Berosus consobrinus

XML Treatment for Berosus corozo

XML Treatment for Berosus ebeninus

XML Treatment for Berosus elegans

XML Treatment for Berosus erraticus

XML Treatment for Berosus festivus

XML Treatment for Berosus garciai

XML Treatment for Berosus geayi

XML Treatment for Berosus ghanicus

XML Treatment for Berosus guyanensis

XML Treatment for Berosus holdhausi

XML Treatment for Berosus humeralis

XML Treatment for Berosus jolyi

XML Treatment for Berosus llanensis

XML Treatment for Berosus marquardti

XML Treatment for Berosus megaphallus

XML Treatment for Berosus olivae

XML Treatment for Berosus ornaticollis

XML Treatment for Berosus pallipes

XML Treatment for Berosus patruelis

XML Treatment for Berosus pluripunctatus

XML Treatment for Berosus reticulatus

XML Treatment for Berosus ruffinus

XML Treatment for Berosus repertus

XML Treatment for Berosus tramidrum

XML Treatment for Berosus truncatipennis

XML Treatment for Berosus wintersteineri

XML Treatment for Berosus zimmermanni

XML Treatment for Berosus sp. A
